# Supplementary material for: Sterols and Triterpenes from Dobera glabra Growing in Saudi Arabia and Their Cytotoxic Activity
Source: Plants (Basel). 2021 Jan 8;10(1):119. doi: 10.3390/plants10010119 (PMC7827382; doi:10.3390/plants10010119)
Supplement: Supplementary file 1 [file plants-10-00119-s001.pdf]

Sterols and Triterpenes from *Dobera glabra* Growing in Saudi Arabia and their Cytotoxic Activity

Wael M. Abdel-Mageed [1,2], Ali A. El-Gamal [1,3,*], Shaza M. Al-Massarani [1], Omer. A. Basudan [1], Farid A. Badria [3], Maged S. Abdel-Kader [4,5], Adnan J. Al-Rehaily [1] and Hanan Y. Al-Ati [1]

[1] Department of Pharmacognosy, College of Pharmacy, King Saud University, P.O. Box 2457, Riyadh 11451, Saudi Arabia; wabdelmageed@ksu.edu.sa (W.M.A.-M.); salmassarani@ksu.edu.sa (S.M.A.-M.); basudan@ksu.edu.sa (O.A.B.); ajhmkl@hotmail.com (A.J.A.-R.); hati@ksu.edu.sa (H.Y.A.-A.)

[2] Pharmacognosy Department, Faculty of Pharmacy, Assiut University, Assiut 71526, Egypt

[3] Department of Pharmacognosy, Faculty of Pharmacy, Mansoura University, El-Mansoura 35516, Egypt; faridbadria@gmail.com

[4] Pharmacognosy Department, College of Pharmacy, Sattam Bin Abdulaziz University, Al-kharj 11942, Saudi Arabia; mpharm101@hotmail.com

[5] Department of Pharmacognosy, College of Pharmacy, Alexandria University, Alexandria 21215, Egypt

* Correspondence: aelgamel@ksu.edu.sa; Tel: +00-9665-6978-0176

E-mail address: aelgamel@ksu.edu.sa (A. A. El-Gamal).

Abstract: A new lupane caffeoyl ester, lup-20(29)-ene 3β-caffeate-30-al (**7**), and a new oleanane-type triterpene, 3*β*-hydroxyolean-13(18)-en-12-one (**17**), were isolated from the aerial parts of *Dobera glabra* (Forssk), along with ten known triterpenes, including seven lupane-type lupeol (**1**), 30-nor-lup-3*β*-ol-20-one (**2**), Δ^1-lupenone (**3**), lup-20(29)-en-3*β*,30-diol (**4**), lupeol caffeate (**5**), 30-hydroxy lup-20(29)-ene 3*β*-caffeate (**6**), and betunaldehyde (**8**); three oleanane-type compounds were also identified, comprising *δ*-amyrone (**15**), *δ*-amyrin (**16**), and 11-oxo-*β*-amyrin (**18**); together with six sterols, comprising *β*-sitosterol (**9**), stigmasterol (**10**), 7*α*-hydroxy-*β*-sitosterol (**11**), 7*α*-hydroxy-stigmasterol (**12**), 7-keto-*β*-sitosterol (**13**), and 7-keto-stigmasterol (**14**). Their structures were elucidated using a variety of spectroscopic techniques, including 1D (^{1}H, ^{13}C, and DEPT-135 ^{13}C) and 2D (^{1}H–^{1}H COSY, ^{1}H–^{13}C HSQC, and ^{1}H–^{13}C HMBC) nuclear magnetic resonance (NMR) and accurate mass spectroscopy. Subsequently, the different plant extracts and some of the isolated compounds (**1–9**, **11** and **13**) were investigated for their possible cytotoxic activity in comparison to cisplatin against a wide array of aggressive cancer cell lines, such as colorectal cancer (HCT-116), hepatocellular carcinoma (HepG-2), and prostate cancer (PC-3) cell lines. Compound **11** displayed broad cytotoxicity against all of the tested cell lines ($IC_{50} \cong 8$ µg/mL in all cases), and a high safety margin against normal *Vero* cells ($IC_{50} = 70$ µg/mL), suggesting that **11** may be a highly selective and effective anticancer agent candidate. Notably, the evidence indicated that the mode of action of compound **11** could possibly consist of the inhibition of phosphodiesterase I (80.2% enzyme inhibition observed at 2 µM compound concentration).

Keywords: *Dobera glabra*; Salvadoraceae; Triterpenes; Steroids, Cytotoxic activity, Phosphodiesterase inhibition.

Content

Content	Page
**Figure 1S.** Structures of compounds (**1–18**) isolated from *D. glabra*.	04
**Figure 2S.** ^{1}H NMR spectrum of compound (**1**) (500 MHz, $CDCl_3$)	05
**Figure 3S.** ^{13}C NMR spectrum of compound (**1**) (125 MHz, $CDCl_3$)	05
**Figure 4S.** DEPT ^{13}C NMR spectrum of compound (**1**) (125 MHz, $CDCl_3$)	06
**Figure 5S.** ^{1}H NMR spectrum of compound (**2**) (500 MHz, $CDCl_3$)	07
**Figure 6S.** ^{13}C NMR spectrum of compound (**2**) (125 MHz, $CDCl_3$)	07
**Figure 7S.** DEPT ^{13}C NMR spectrum of compound (**2**) (125 MHz, $CDCl_3$)	08
**Figure 8S.** ^{1}H NMR spectrum of compound (**3**) (500 MHz, $CDCl_3$)	09
**Figure 9S.** ^{13}C NMR spectrum of compound (**3**) (125 MHz, $CDCl_3$)	09
**Figure 10S.** DEPT ^{13}C NMR spectrum of compound (**3**) (125 MHz, $CDCl_3$)	10
**Figure 11S.** ^{1}H NMR spectrum of compound (**4**) (500 MHz, $CDCl_3$)	11
**Figure 12S.** ^{13}C NMR spectrum of compound (**4**) (125 MHz, $CDCl_3$)	11
**Figure 13S.** ^{1}H NMR spectrum of compound (**5**) (500 MHz, $CDCl_3$)	12
**Figure 14S.** ^{13}C NMR spectrum of compound (**5**) (125 MHz, $CDCl_3$)	12
**Figure 15S.** DEPT ^{13}C NMR spectrum of compound (**5**) (125 MHz, $CDCl_3$)	13
**Figure 16S:** ^{1}H NMR spectrum of compound (**6**) (500 MHz, $CDCl_3$)	14
**Figure 17S.** ^{13}C NMR spectrum of compound (**6**) (125 MHz, $CDCl_3$)	14
**Figure 18S.** DEPT ^{13}C NMR spectrum of compound (**6**) (125 MHz, $CDCl_3$)	15
**Figure 19S.** ^{1}H NMR spectrum of compound (**7**) (500 MHz, $CDCl_3$)	16
**Figure 20S.** ^{13}C NMR spectrum of compound (**7**) (125 MHz, $CDCl_3$)	16
**Figure 21S.** DEPT ^{13}C NMR spectrum of compound (**7**) (125 MHz, $CDCl_3$)	17
**Figure 22S.** ^{1}H-^{13}C HSQC spectrum of compound (**7**) (500 MHz, $CDCl_3$)	17
**Figure 23S.** ^{1}H-^{1}H COSY spectrum of compound (**7**) (500 MHz, $CDCl_3$)	18
**Figure 24S:** ^{1}H-^{13}C HMBC spectrum of compound (**7**) (500 MHz, $CDCl_3$)	18
**Figure 25S:** HRESIMS spectrum of compound (**7**) (A) negative mode, (B) positive mode.	19
**Figure 26S:** ^{1}H NMR spectrum of compound (**8**) (500 MHz, $DMSO_6$)	20
**Figure 27S.** ^{13}C NMR spectrum of compound (**8**) (125 MHz, $DMSO_6$)	20
**Figure 28S.** DEPT ^{13}C NMR spectrum of compound (**8**) (125 MHz, $DMSO_6$)	21
**Figure 29S.** ^{1}H NMR spectrum of compound (**13**) (500 MHz, $CDCl_3$)	22
**Figure 30S.** ^{13}C NMR spectrum of compound (**13**) (125 MHz, $CDCl_3$)	22
**Figure 31S.** DEPT ^{13}C NMR spectrum of compound (**13**) (125 MHz, $CDCl_3$)	23
**Figure 32S.** ^{1}H NMR spectrum of compound (**14**) (500 MHz, $CDCl_3$)	24
**Figure 33S.** ^{13}C NMR spectrum of compound (**14**) (125 MHz, $CDCl_3$)	24
**Figure 34S.** DEPT ^{13}C NMR spectrum of compound (**14**) (125 MHz, $CDCl_3$)	25
**Figure 35S.** ^{1}H NMR spectrum of compound (**15**) (500 MHz, $CDCl_3$)	26
**Figure 36S.** ^{13}C NMR spectrum of compound (**15**) (125 MHz, $CDCl_3$)	26
**Figure 37S.** ^{1}H NMR spectrum of compound (**16**) (500 MHz, $CDCl_3$)	27
**Figure 38S.** ^{13}C NMR spectrum of compound (**16**) (125 MHz, $CDCl_3$)	27
**Figure 39S.** ^{1}H NMR spectrum of compound (**17**) (500 MHz, CD_3OD)	28
**Figure 40S.** ^{13}C NMR spectrum of compound (**17**) (125 MHz, CD_3O)	28
**Figure 41S.** DEPT ^{13}C NMR spectrum of compound (**17**) (125 MHz, CD_3OD)	29
**Figure 42S.** ^{1}H-^{13}C HSQC spectrum of compound (**17**) (500 MHz, CD_3OD)	29
**Figure 43S.** ^{1}H-^{1}H COSY spectrum of compound (**17**) (500 MHz, CD_3OD)	30
**Figure 44S.** ^{1}H-^{13}C HMBC spectrum of compound (**17**) (500 MHz, CD3OD)	30
**Figure 45S:** HRESIMS spectrum of compound (**17**) positive mode.	31
**Figure 46S.** ^{1}H NMR spectrum of compound (**18**) (500 MHz, $CDCl_3$)	32
**Figure 47S.** ^{13}C NMR spectrum of compound (**18**) (125 MHz, $CDCl_3$)	32
**Figure 48S.** DEPT ^{13}C NMR spectrum of compound (**18**) (125 MHz, $CDCl_3$)	33

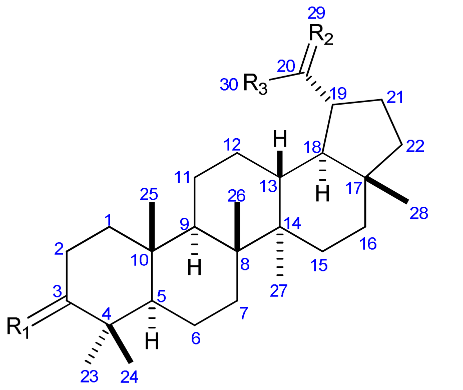

1 R_1= α-H, β-OH, R_2= CH_2, R_3= CH_3
2 R_1= α-H, β-OH, R_2= O, R_3= CH_3
3 R_1= O, $^{1,2}\Delta$, R_2= CH_2, R_3= CH_3
4 R_1= α-H, β-OH, R_2= CH_2, R_3= CH_2OH
5 R_1= caffeoyl, R_2= CH_2, R_3= CH_3
6 R_1= caffeoyl, R_2= CH_2, R_3= CH_2OH
7 R_1= caffeoyl, R_2= CH_2, R_3= CHO
8 R_1= α-H, β-OH, R_2= O, R_3= OH

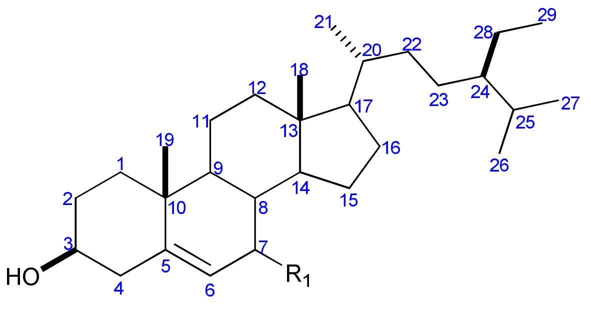

9 R_1= H
10 R_1= H, $^{22,23}\Delta$
11 R_1= OH
12 R_1= OH, $^{22,23}\Delta$
13 R_1= O
14 R_1= O, $^{22,23}\Delta$

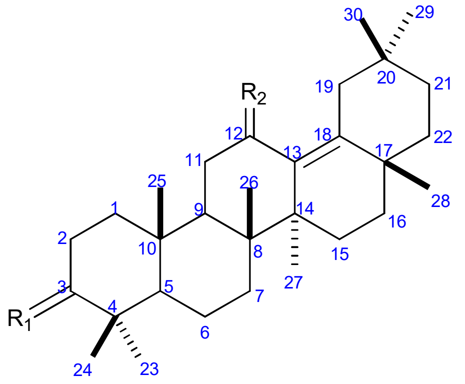

15 R_1= O, R_2= H_2
16 R_1= α-H, β-OH, R_2= H_2
17 R_1= α-H, β-OH, R_2= O

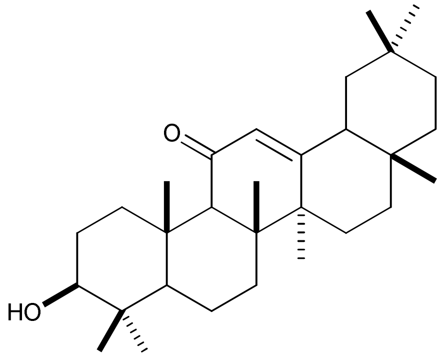

18

Caffeoyl =

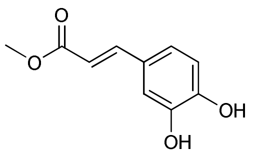

Fig. 1S. Structures of compounds (**1–18**) isolated from *D. glabra.*

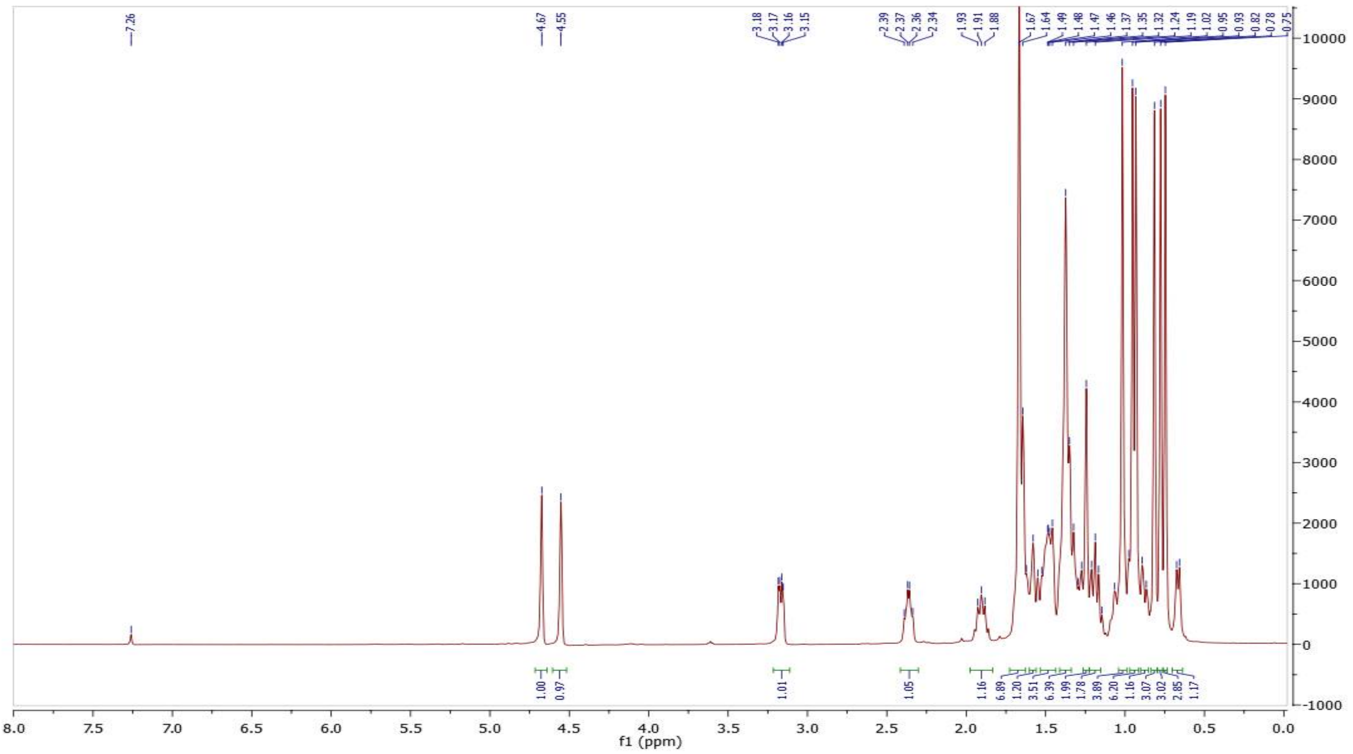

Figure 2S. ^{1}H NMR spectrum of compound (**1**) (500 MHz, $CDCl_3$)

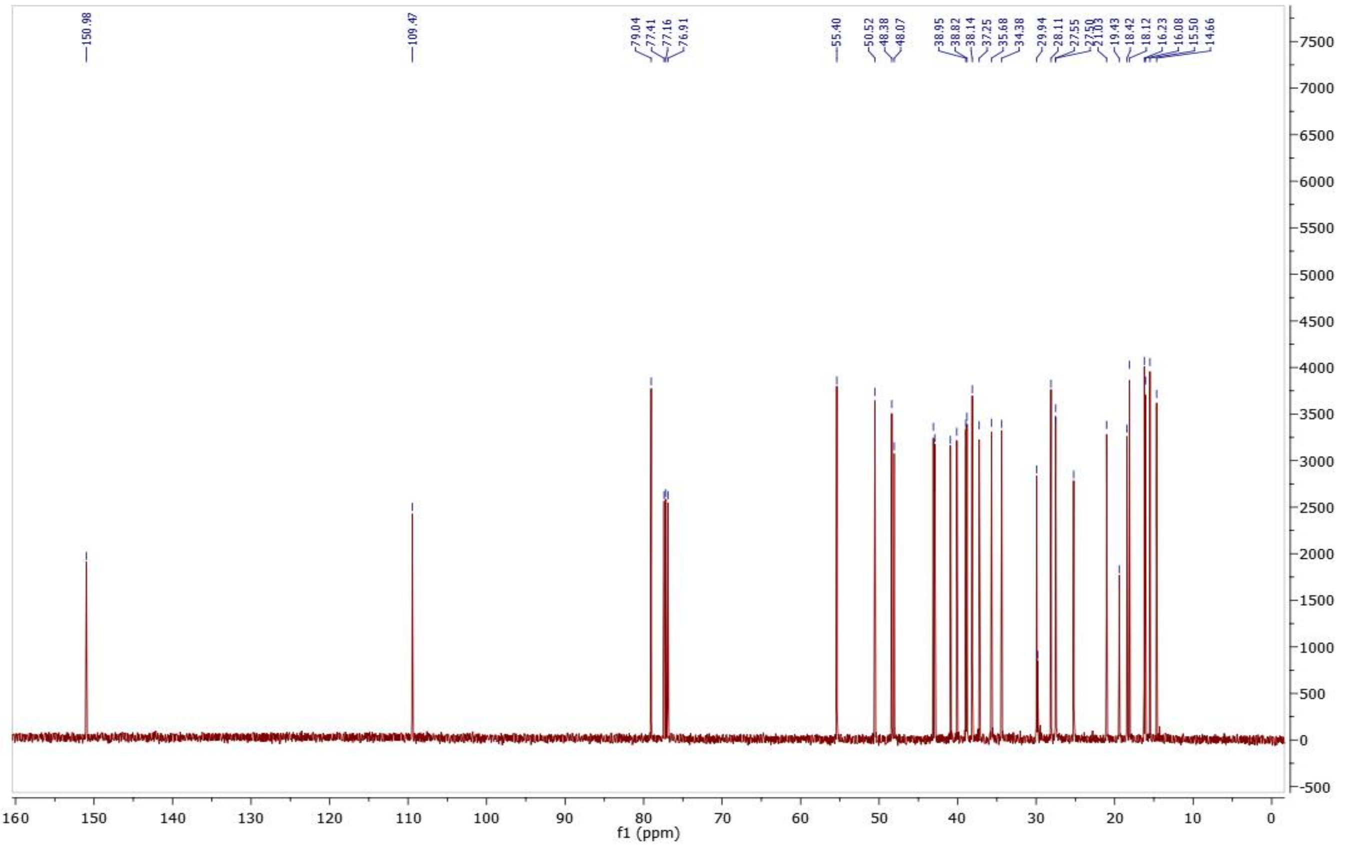

Figure 3S. ^{13}C NMR spectrum of compound (**1**) (125 MHz, $CDCl_3$)

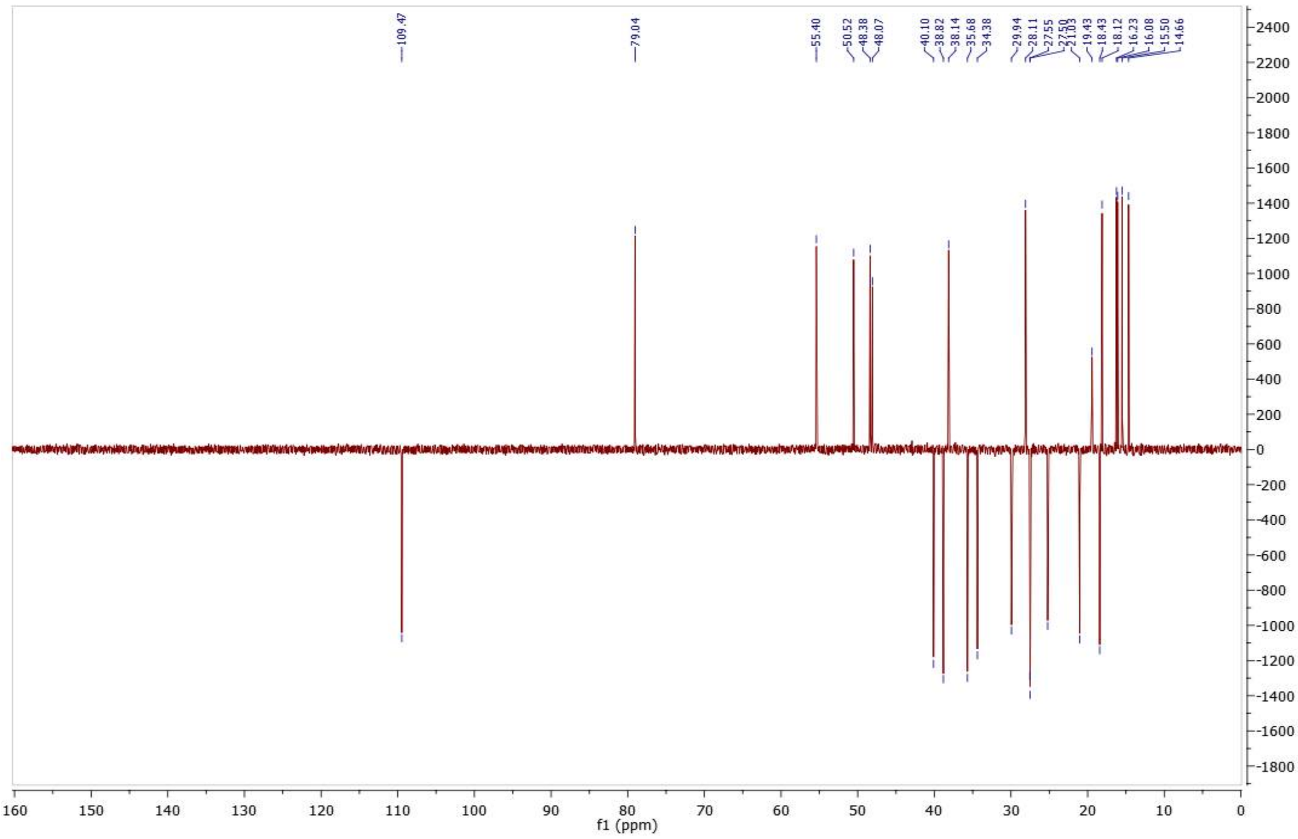

Figure 4S. DEPT ^{13}C NMR spectrum of compound (**1**) (125 MHz, $CDCl_3$)

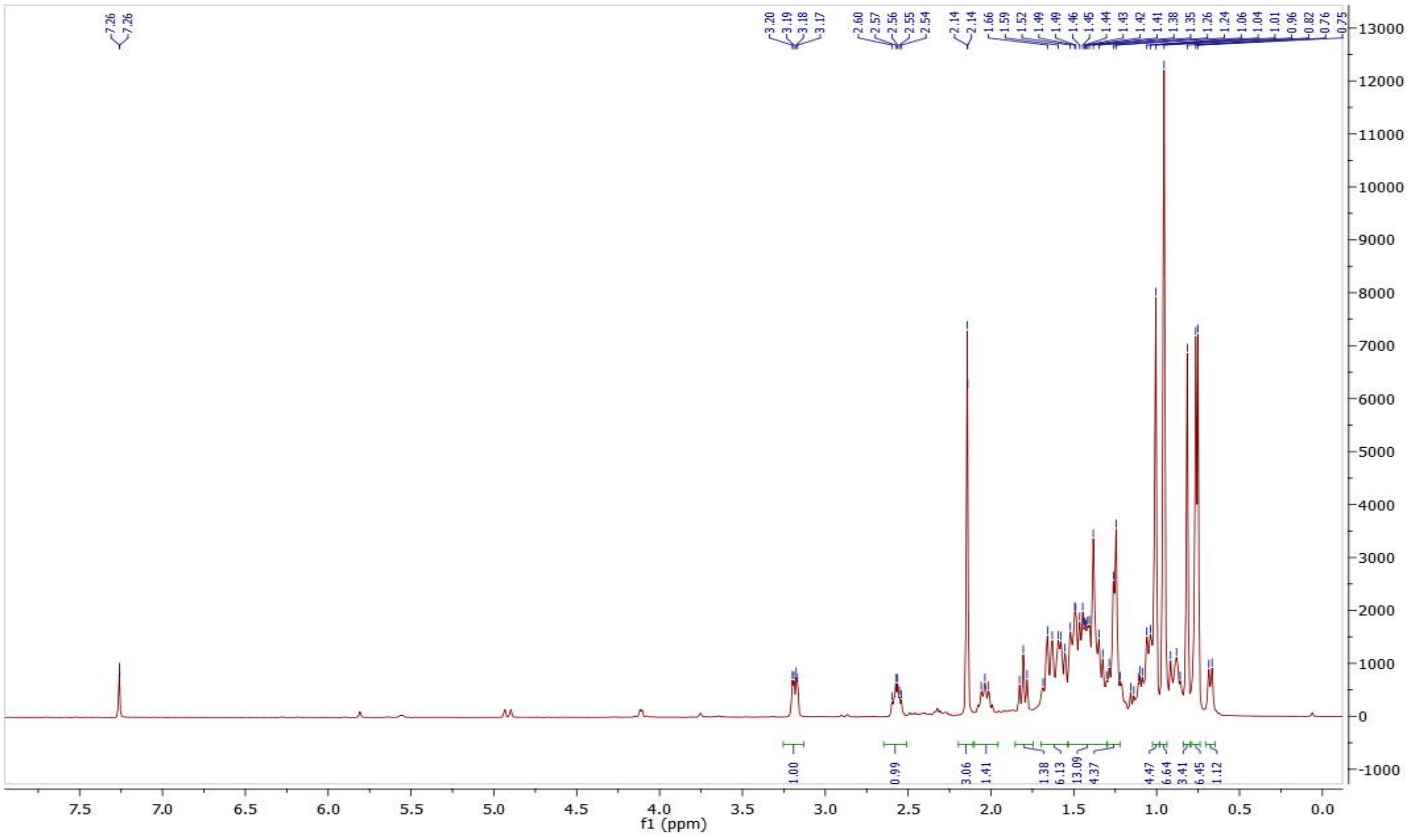

Figure 5S. ^{1}H NMR spectrum of compound (**2**) (500 MHz, $CDCl_3$)

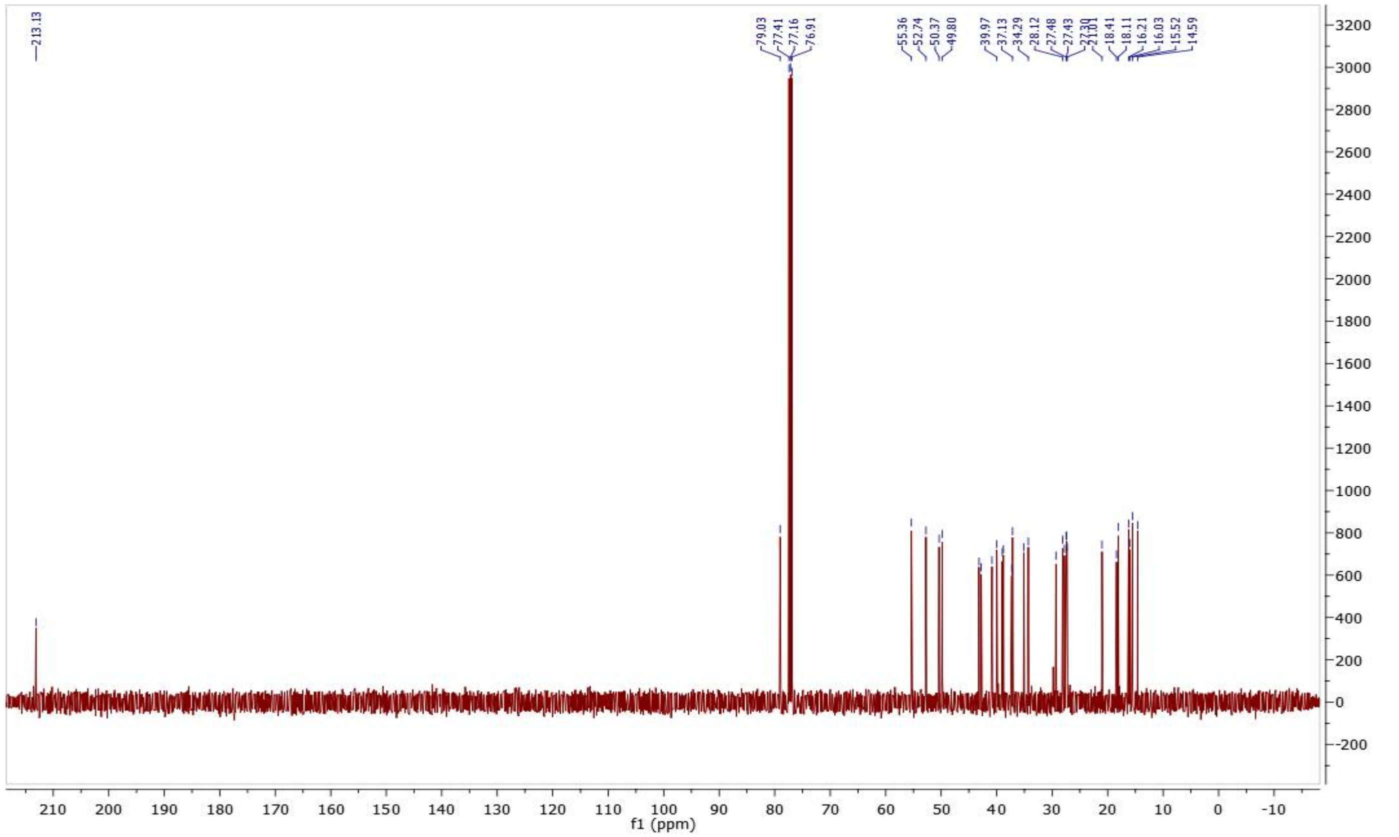

Figure 6S. ^{13}C NMR spectrum of compound (**2**) (125 MHz, $CDCl_3$)

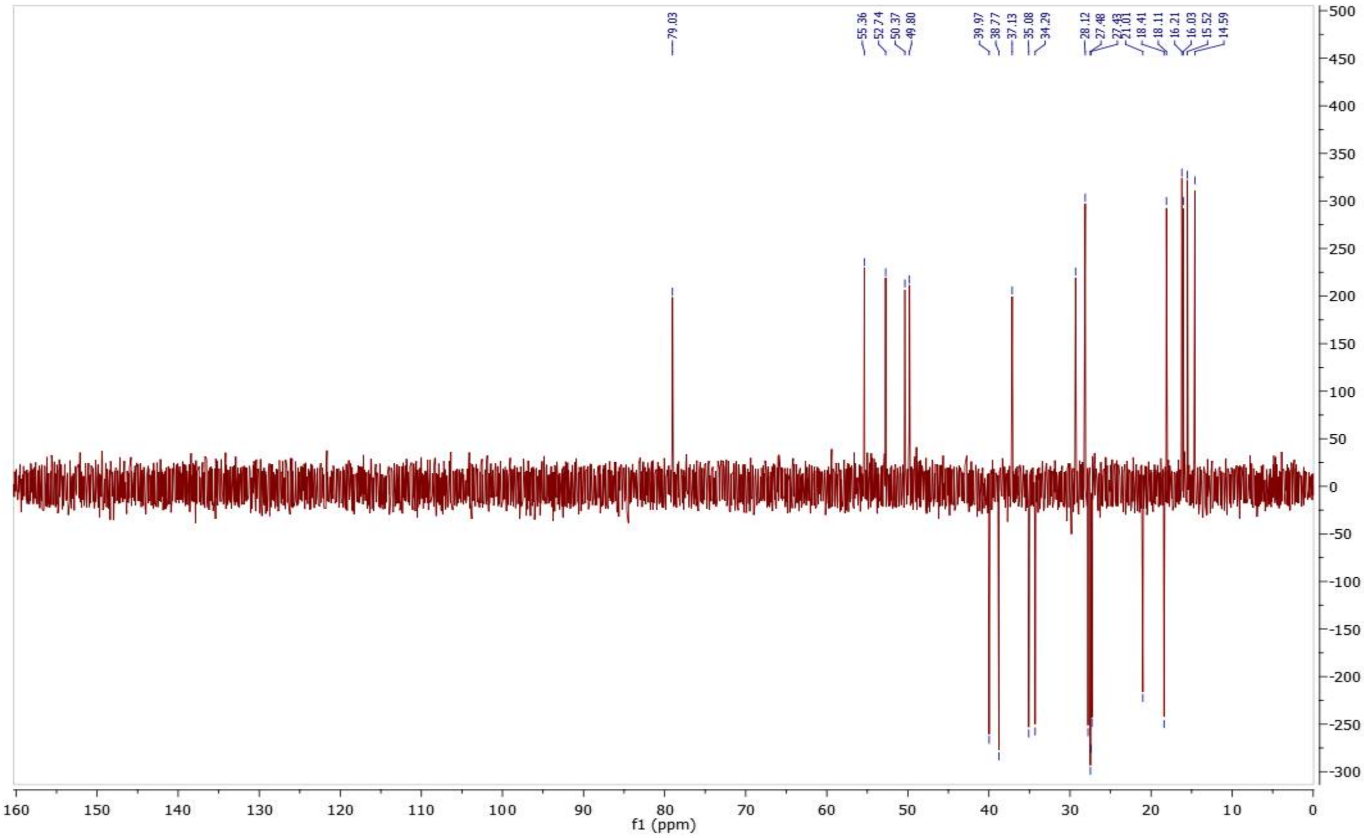

Figure 7S. DEPT ^{13}C NMR spectrum of compound (**2**) (125 MHz, $CDCl_3$)

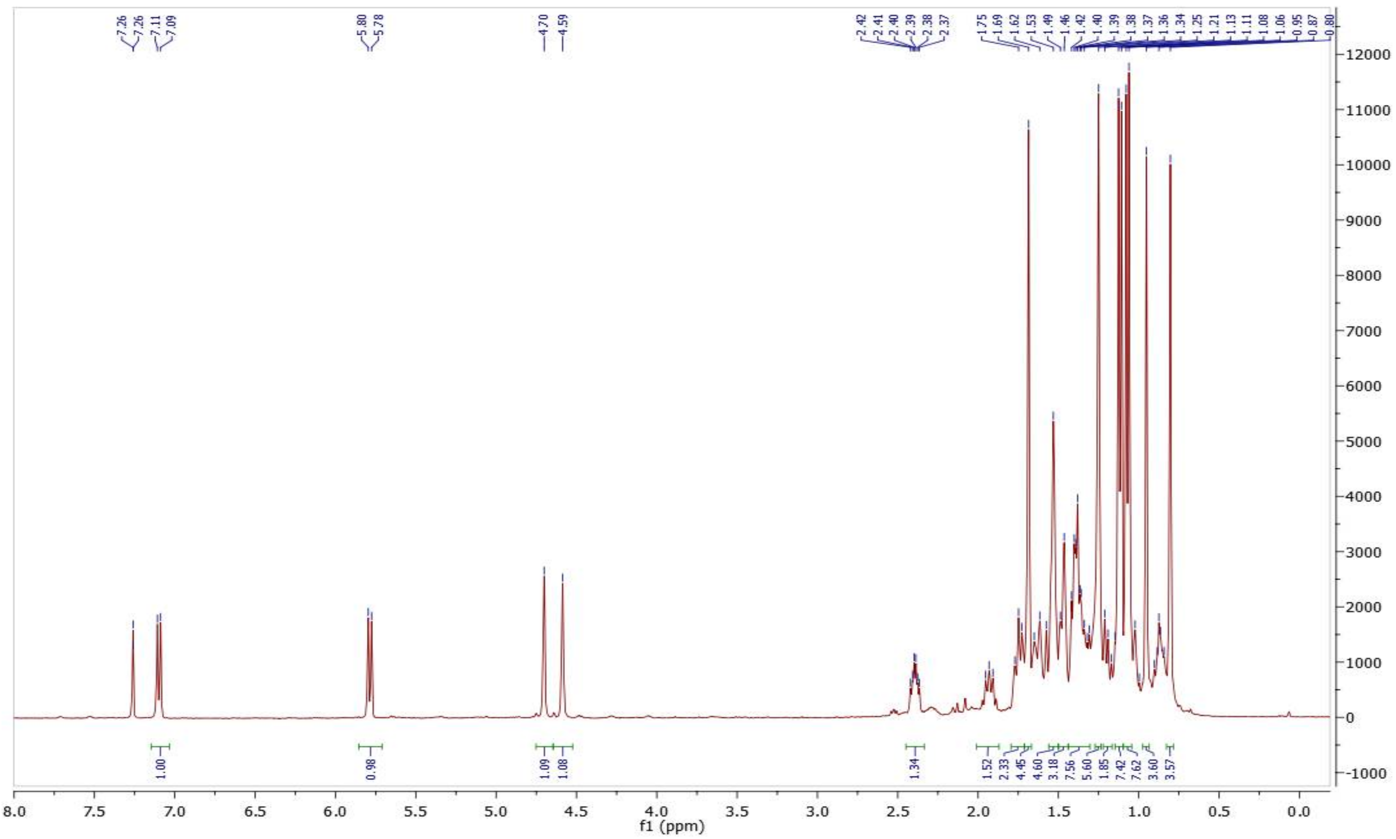

Figure 8S. ^{1}H NMR spectrum of compound (**3**) (500 MHz, $CDCl_3$)

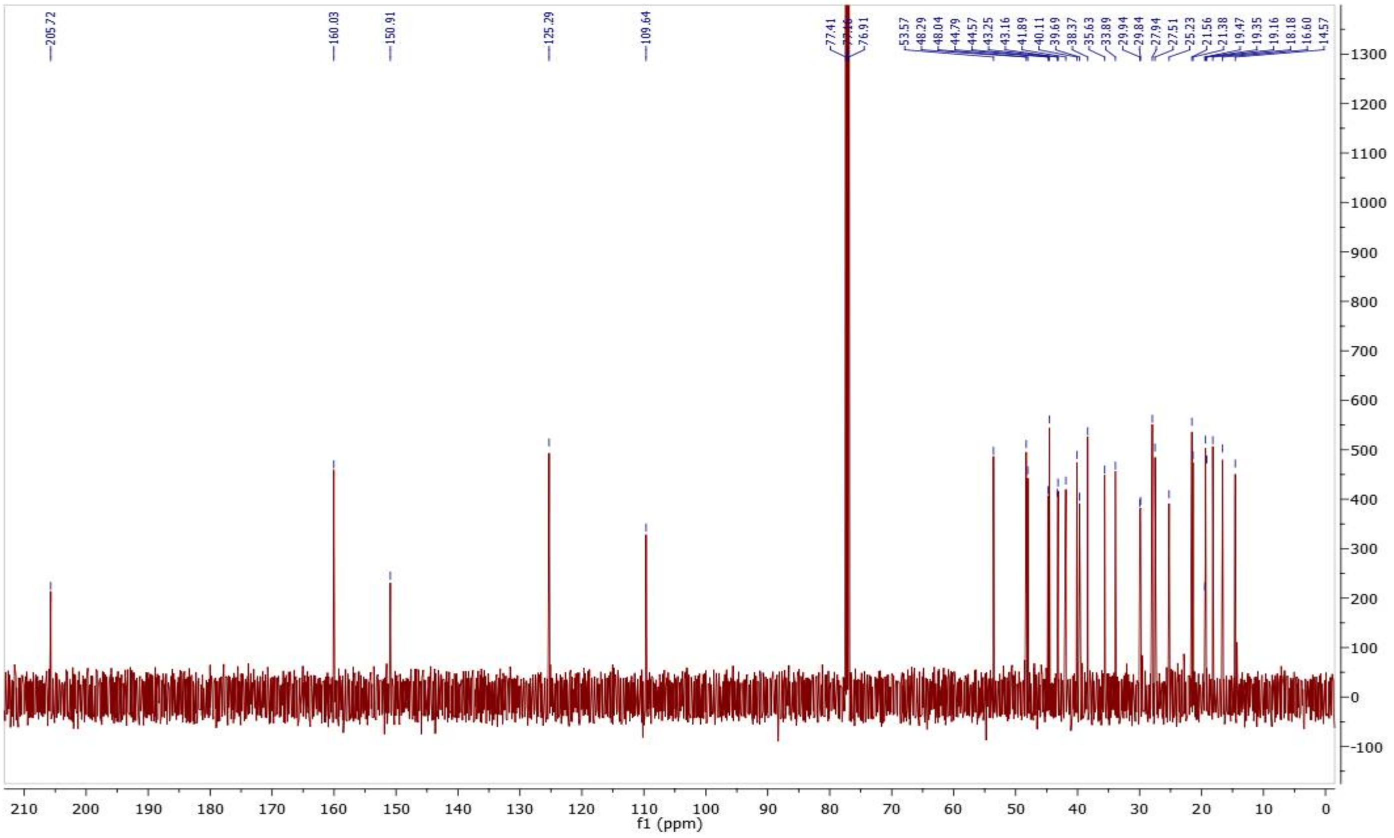

Figure 9S. ^{13}C NMR spectrum of compound (**3**) (125 MHz, $CDCl_3$)

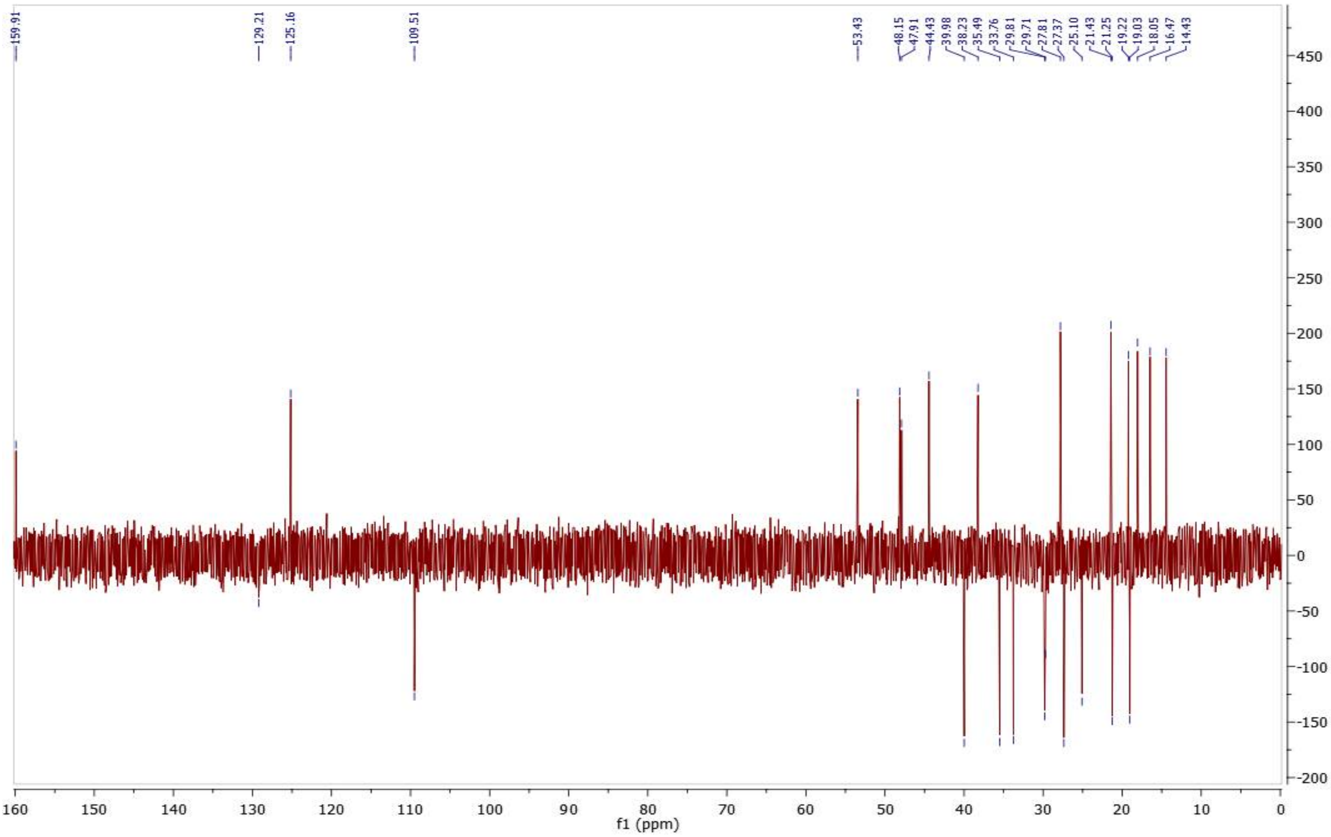

Figure 10S. DEPT ^{13}C NMR spectrum of compound (**3**) (125 MHz, $CDCl_3$)

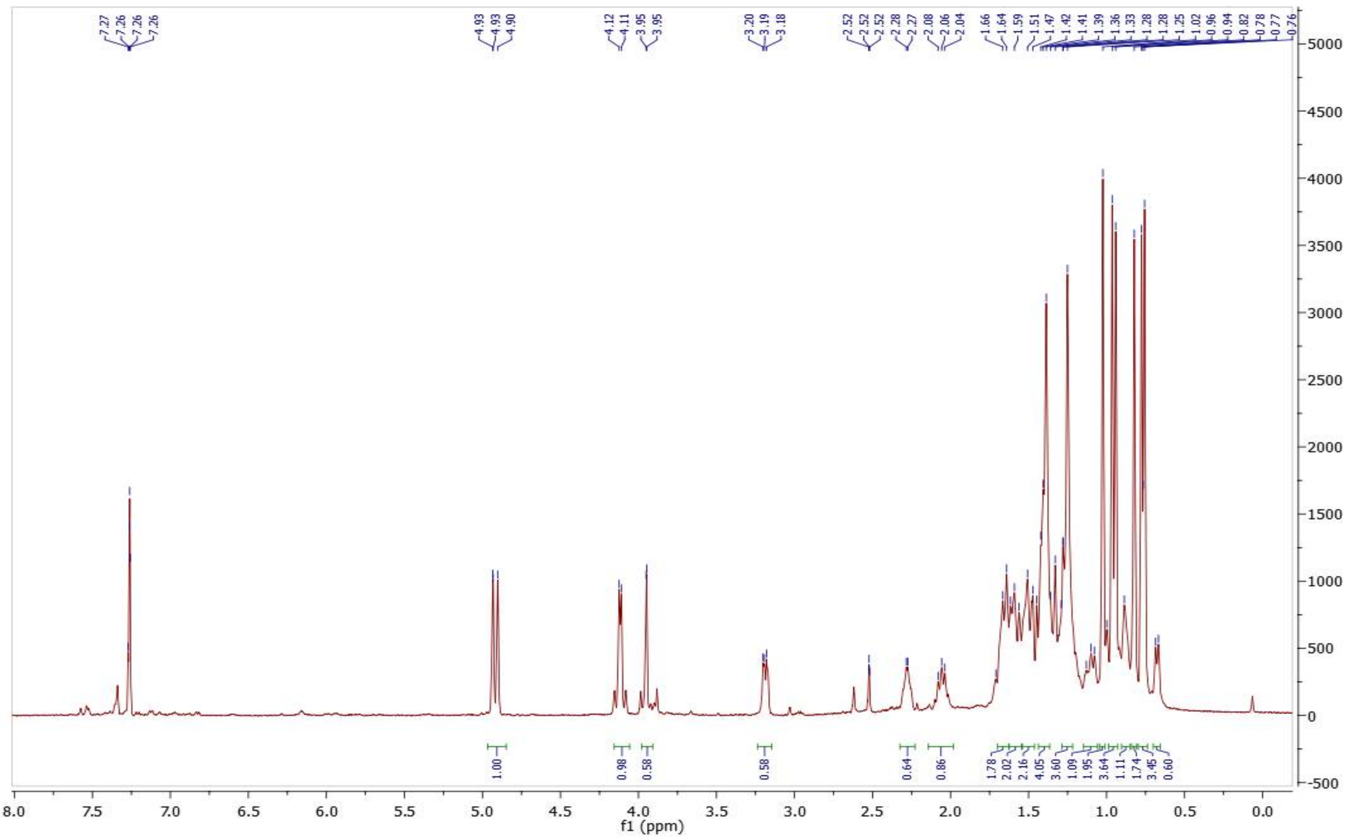

Figure 11S. ^{1}H NMR spectrum of compound (**4**) (500 MHz, $CDCl_3$)

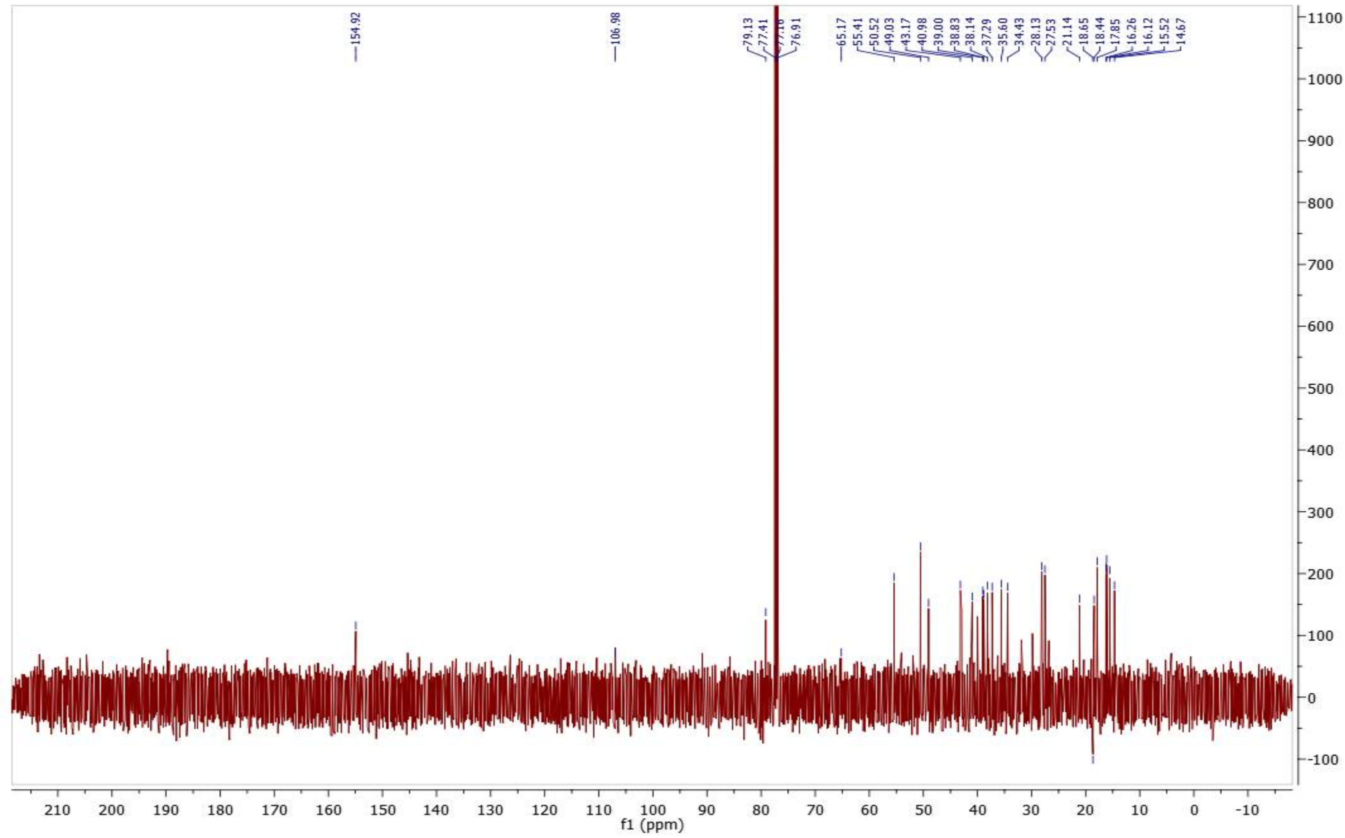

Figure 12S. ^{13}C NMR spectrum of compound (**4**) (125 MHz, $CDCl_3$)

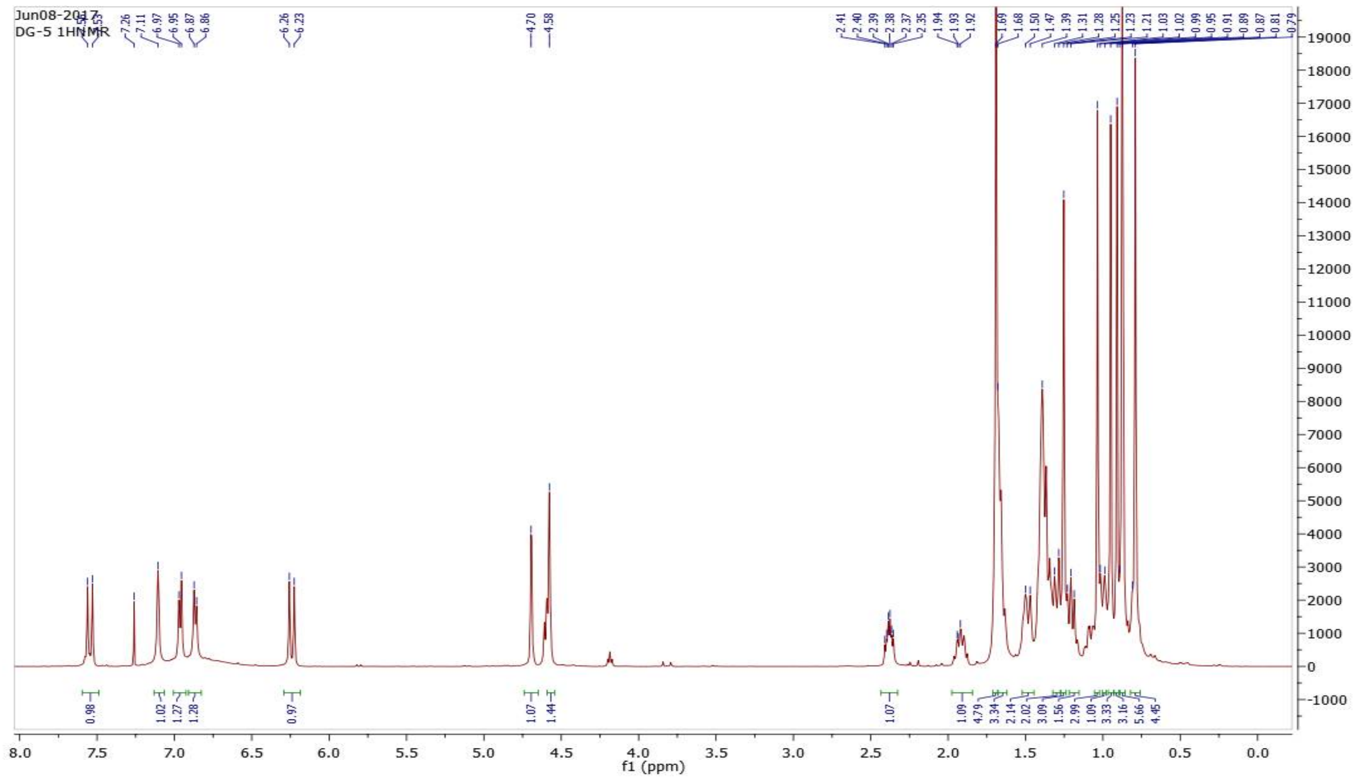

Figure 13S. ^{1}H NMR spectrum of compound (**5**) (500 MHz, $CDCl_3$)

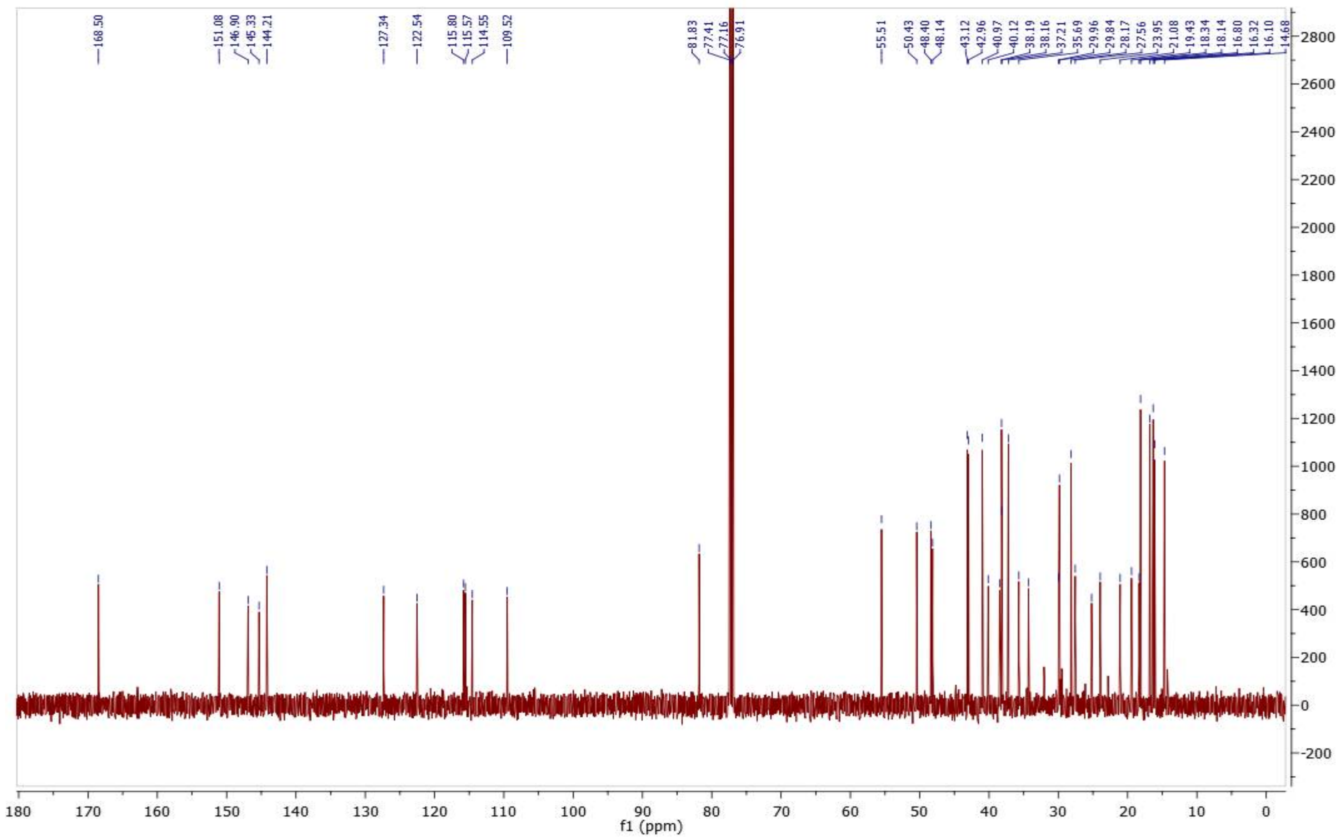

Figure 14S. ^{13}C NMR spectrum of compound (**5**) (125 MHz, $CDCl_3$)

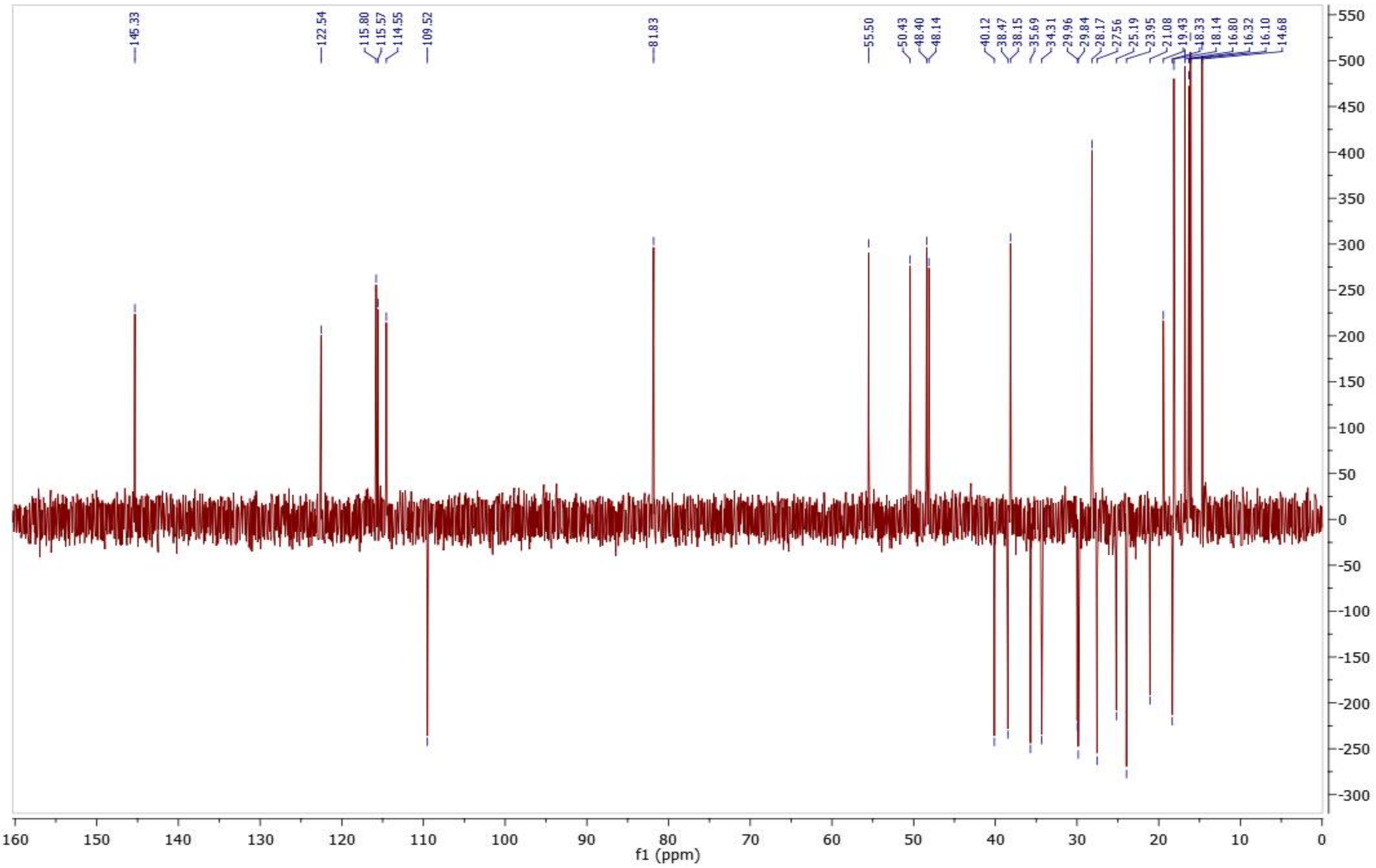

Figure 15S. DEPT ^{13}C NMR spectrum of compound (**5**) (125 MHz, $CDCl_3$)

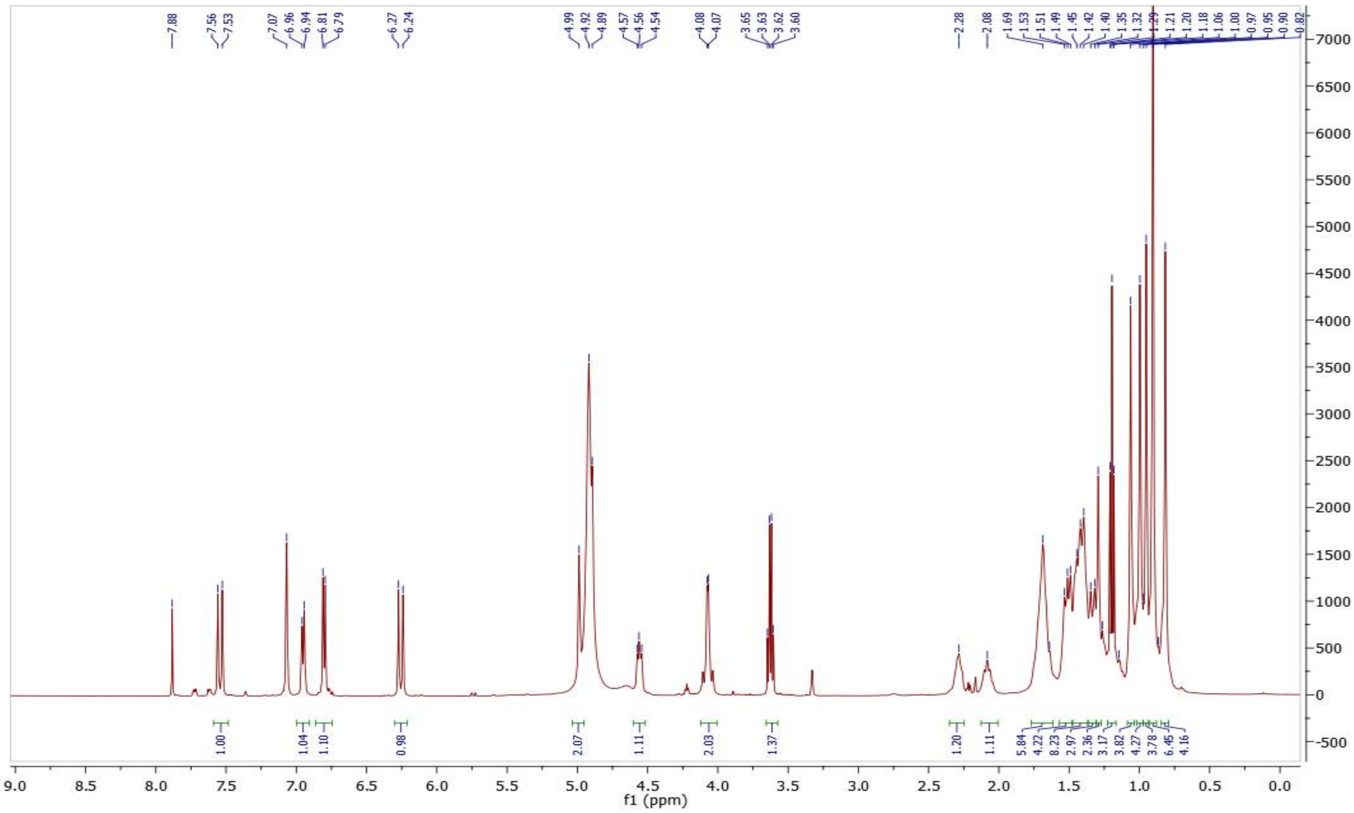

Figure 16S. ^{1}H NMR spectrum of compound (**6**) (500 MHz, $CDCl_3$)

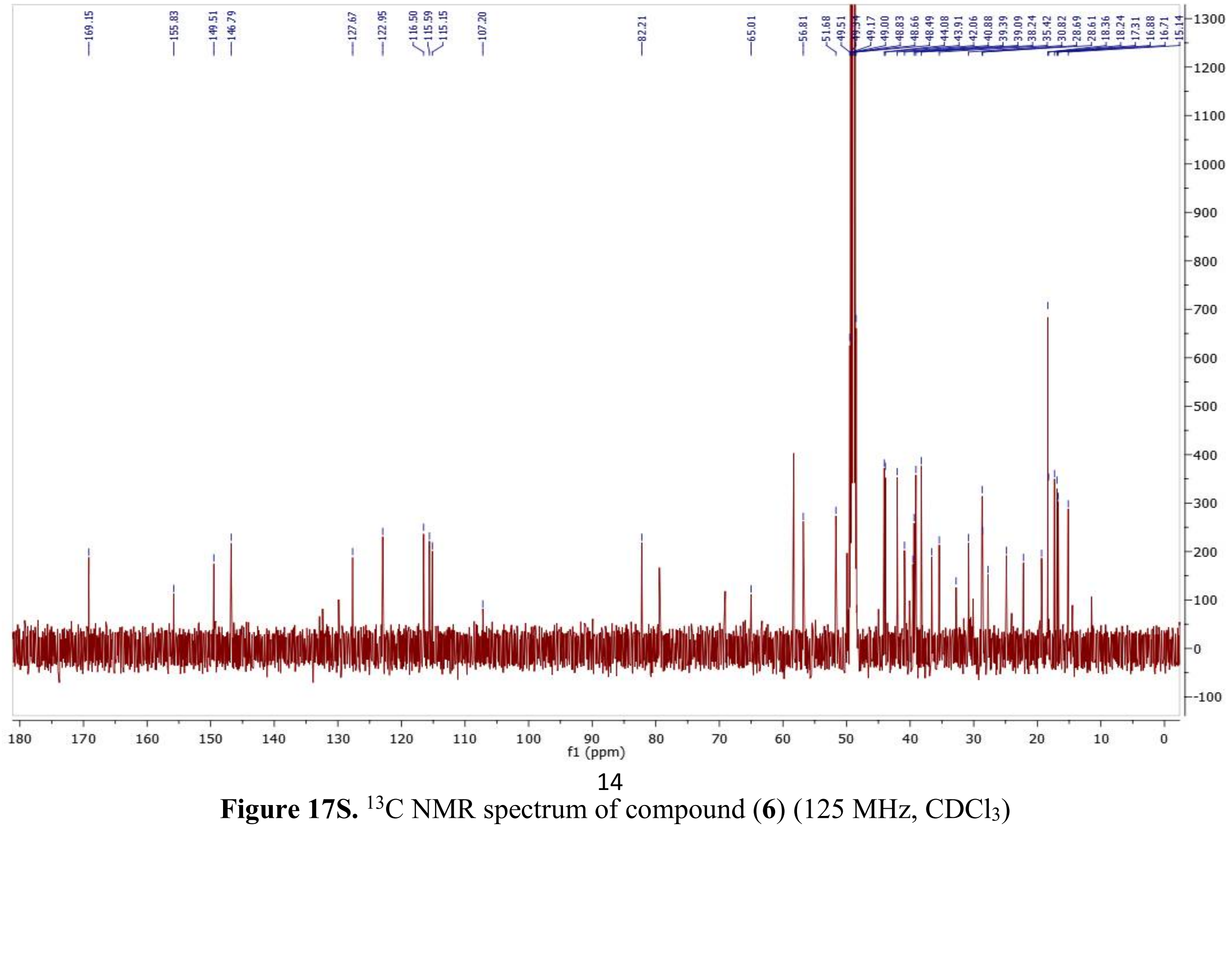

Figure 17S. ^{13}C NMR spectrum of compound (**6**) (125 MHz, $CDCl_3$)

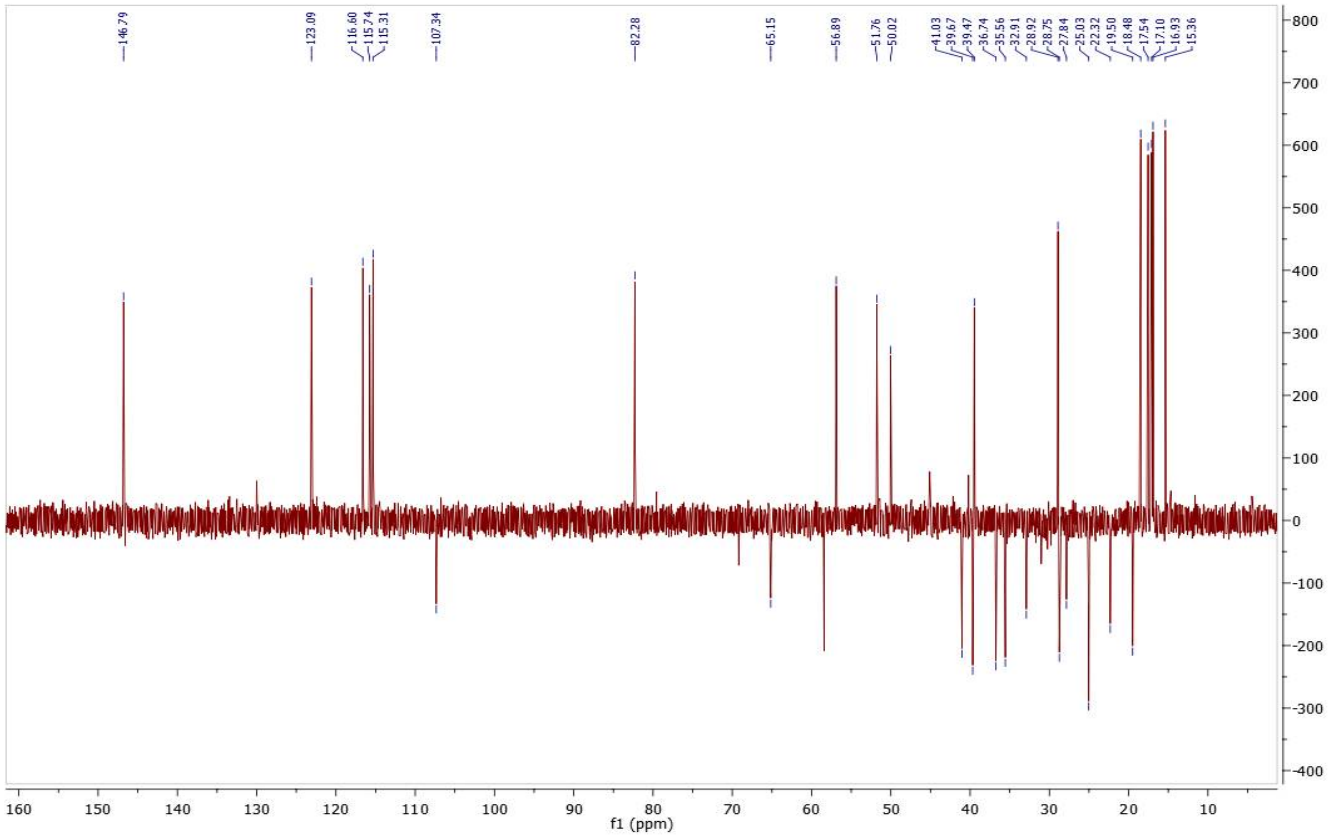

Figure 18S. DEPT ^{13}C NMR spectrum of compound (**6**) (125 MHz, $CDCl_3$)

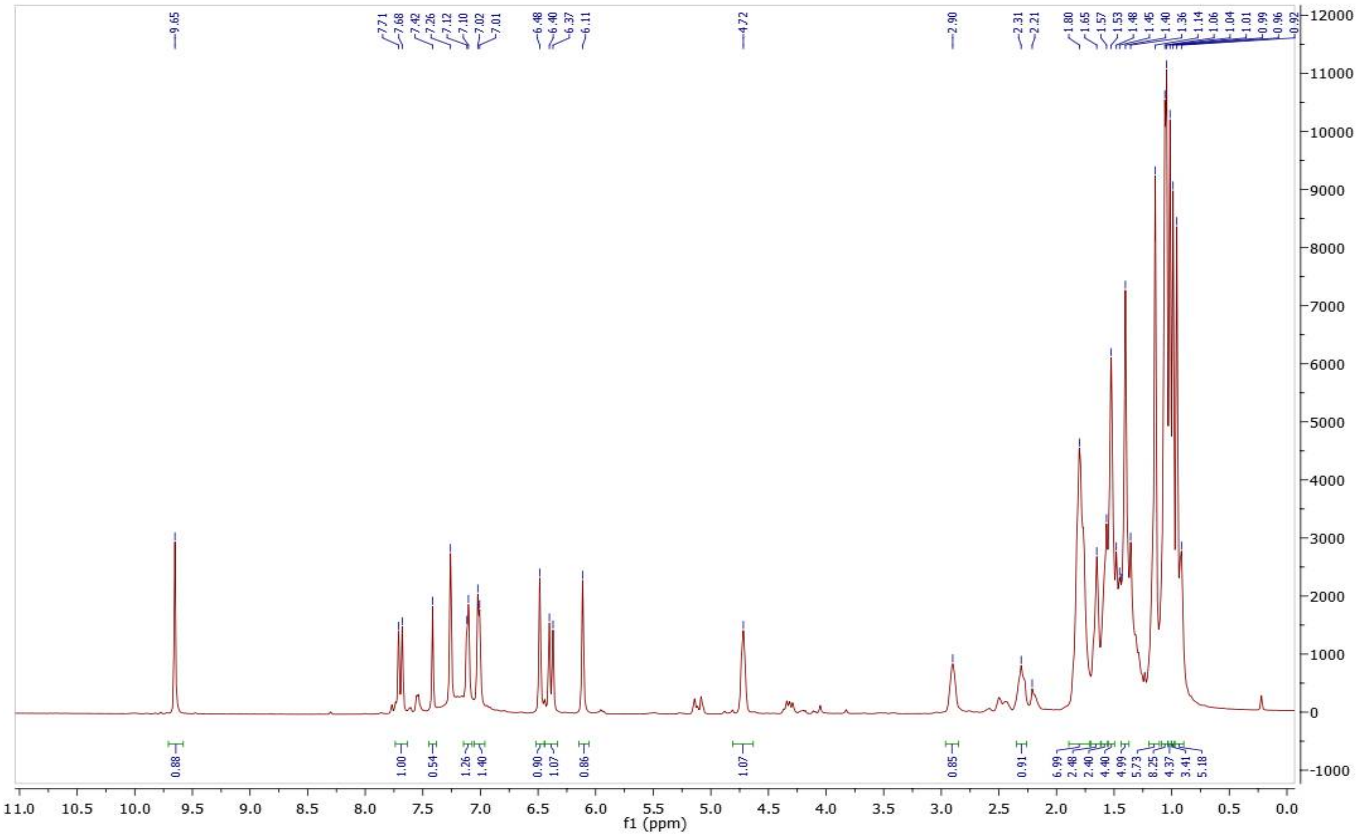

Figure 19S. ^{1}H NMR spectrum of compound (**7**) (500 MHz, $CDCl_3$)

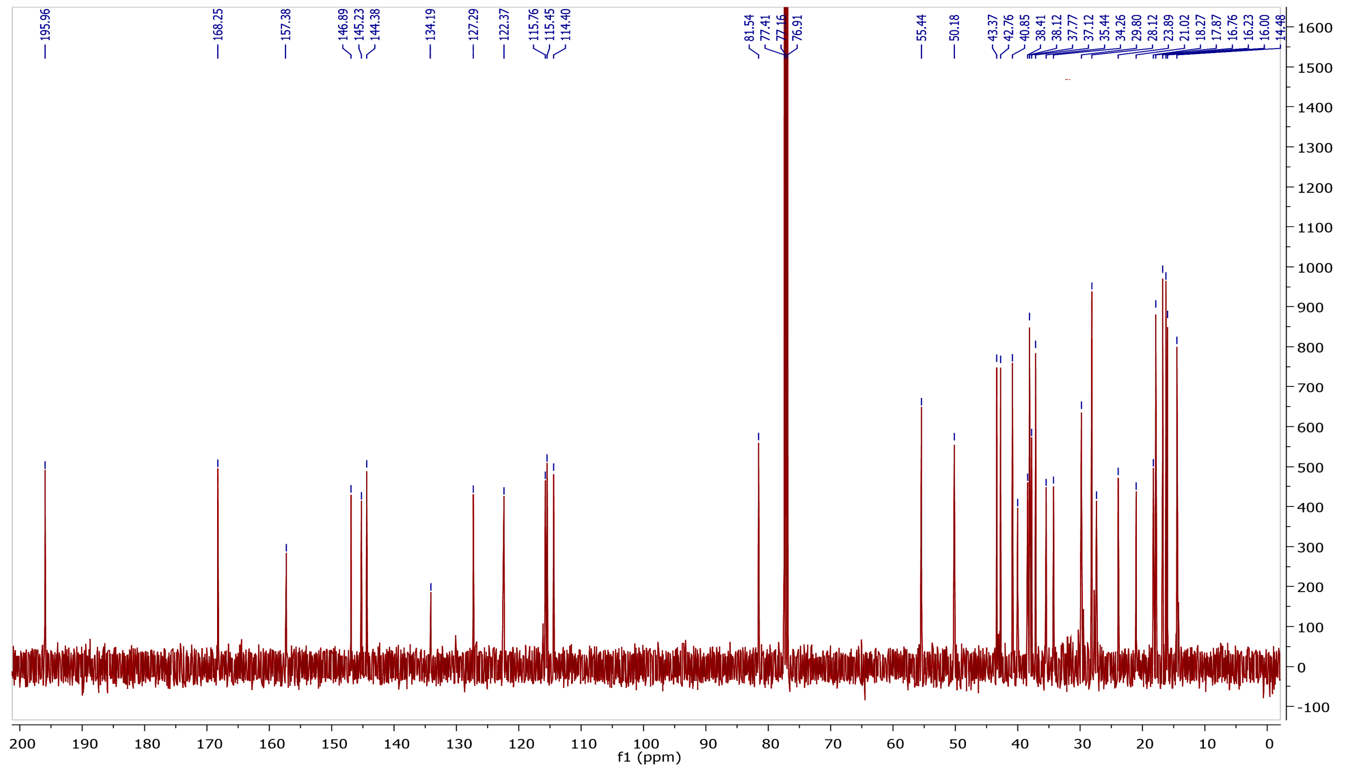

Figure 20S. ^{13}C NMR spectrum of compound (**7**) (125 MHz, $CDCl_3$)

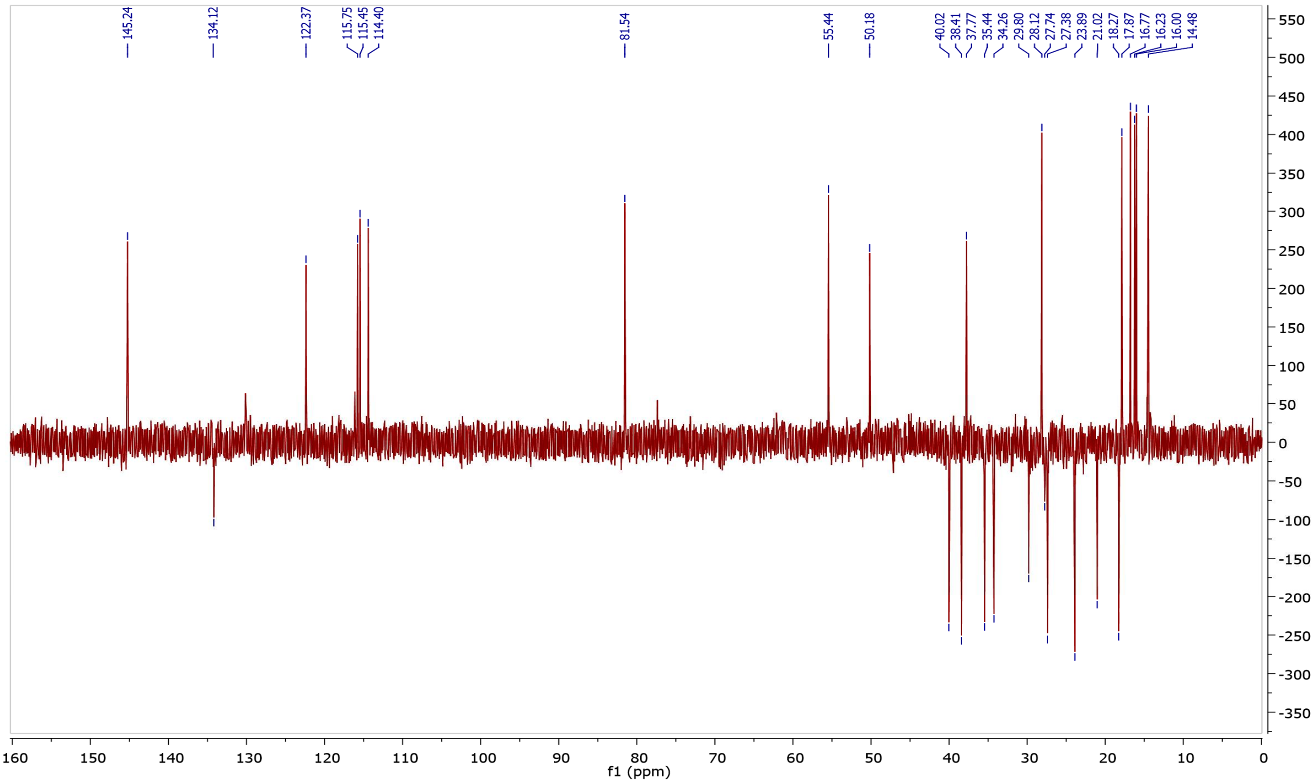

Figure 21S. DEPT ^{13}C NMR spectrum of compound (**7**) (125 MHz, $CDCl_3$)

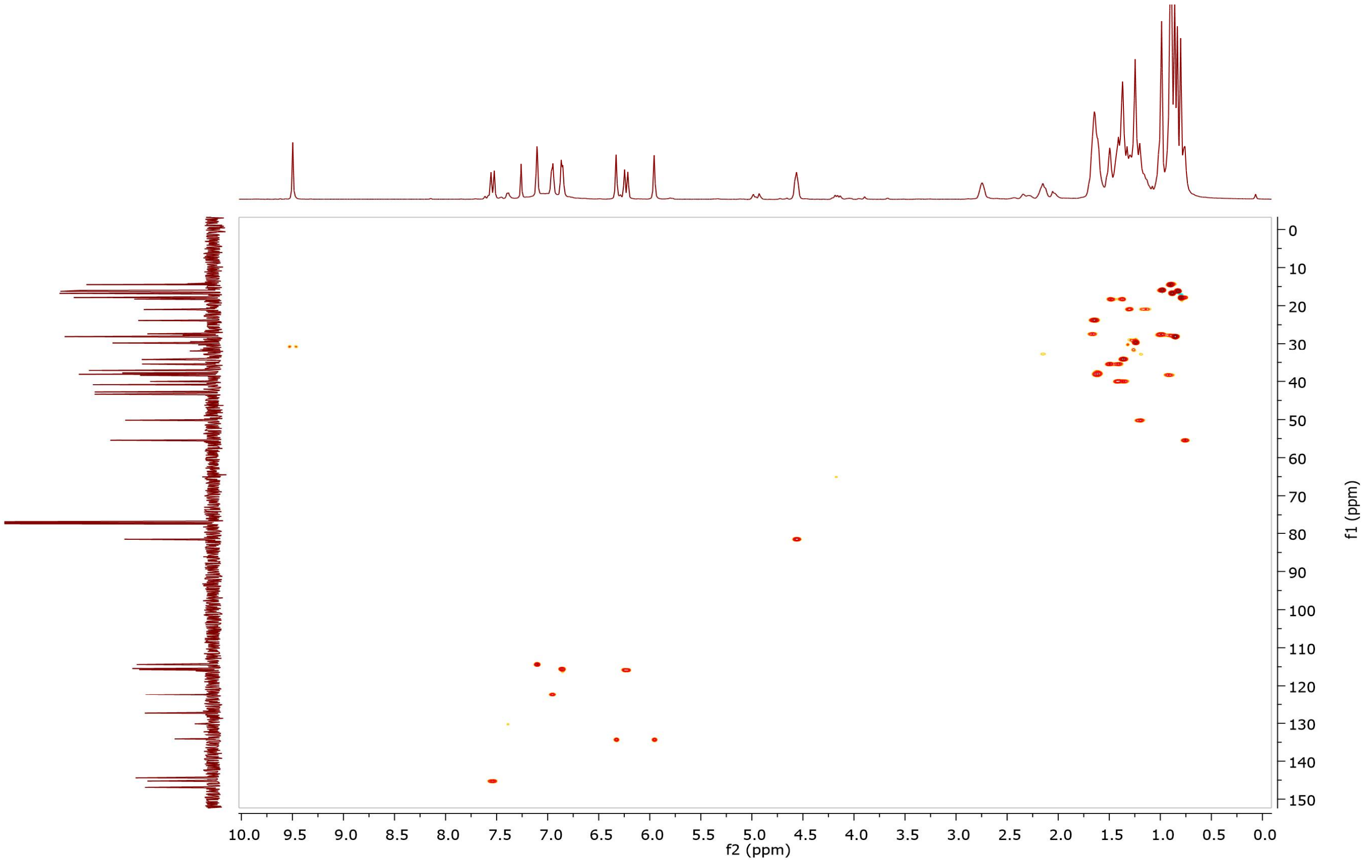

Figure 22S. ^{1}H-^{13}C HSQC spectrum of compound (**7**) (500 MHz, $CDCl_3$)

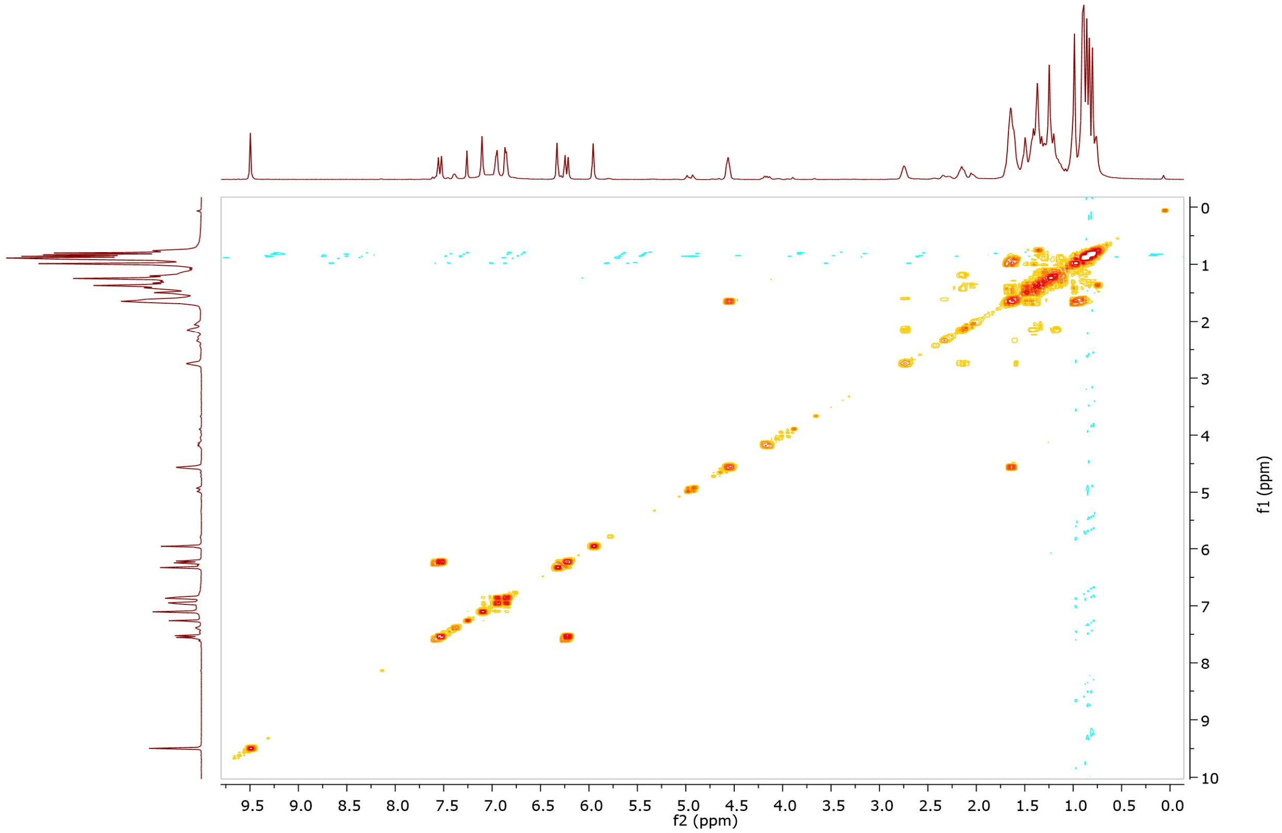

Figure 23S. ^{1}H-^{1}H COSY spectrum of compound (**7**) (500 MHz, $CDCl_3$)

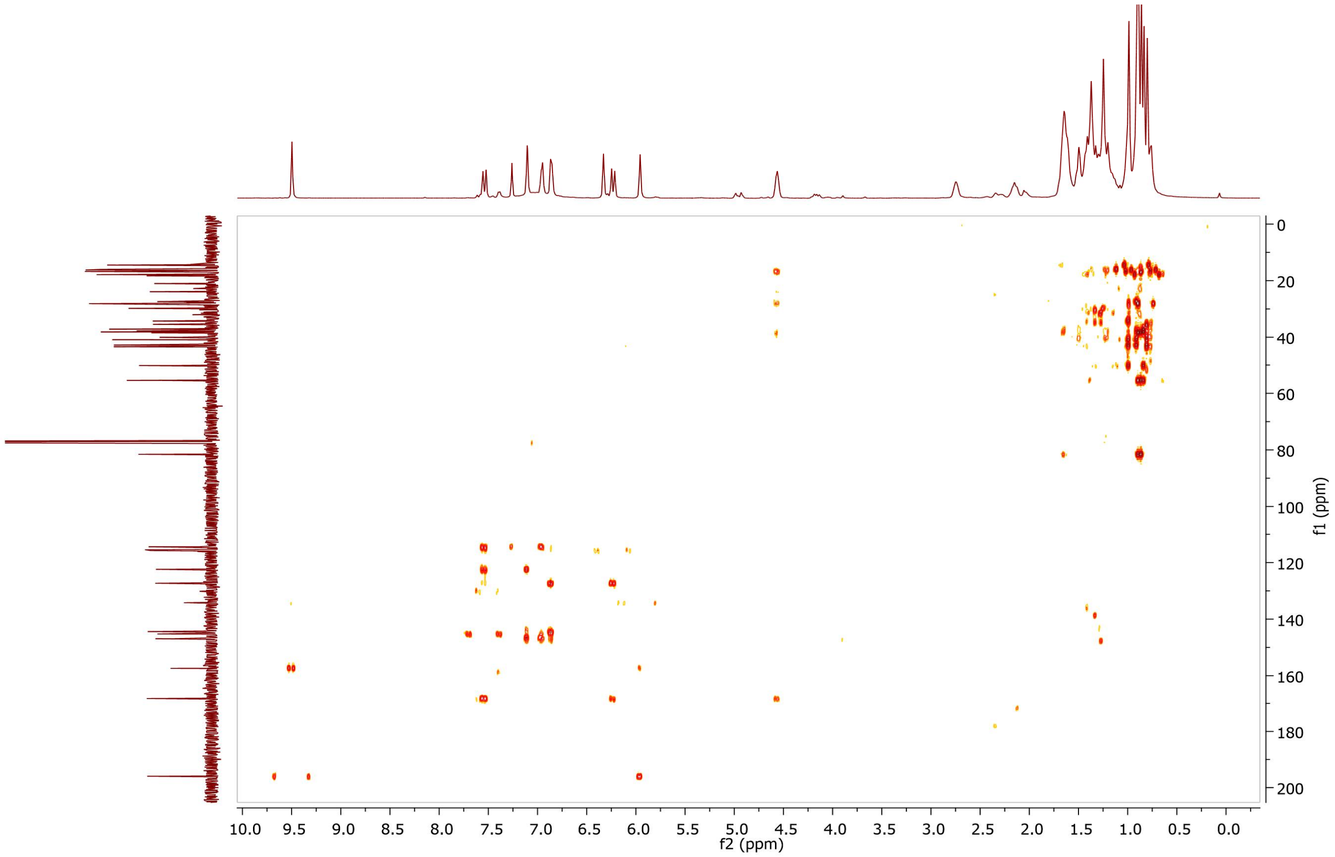

Figure 24S. ^{1}H-^{13}C HMBC spectrum of compound (**7**) (500 MHz, $CDCl_3$)

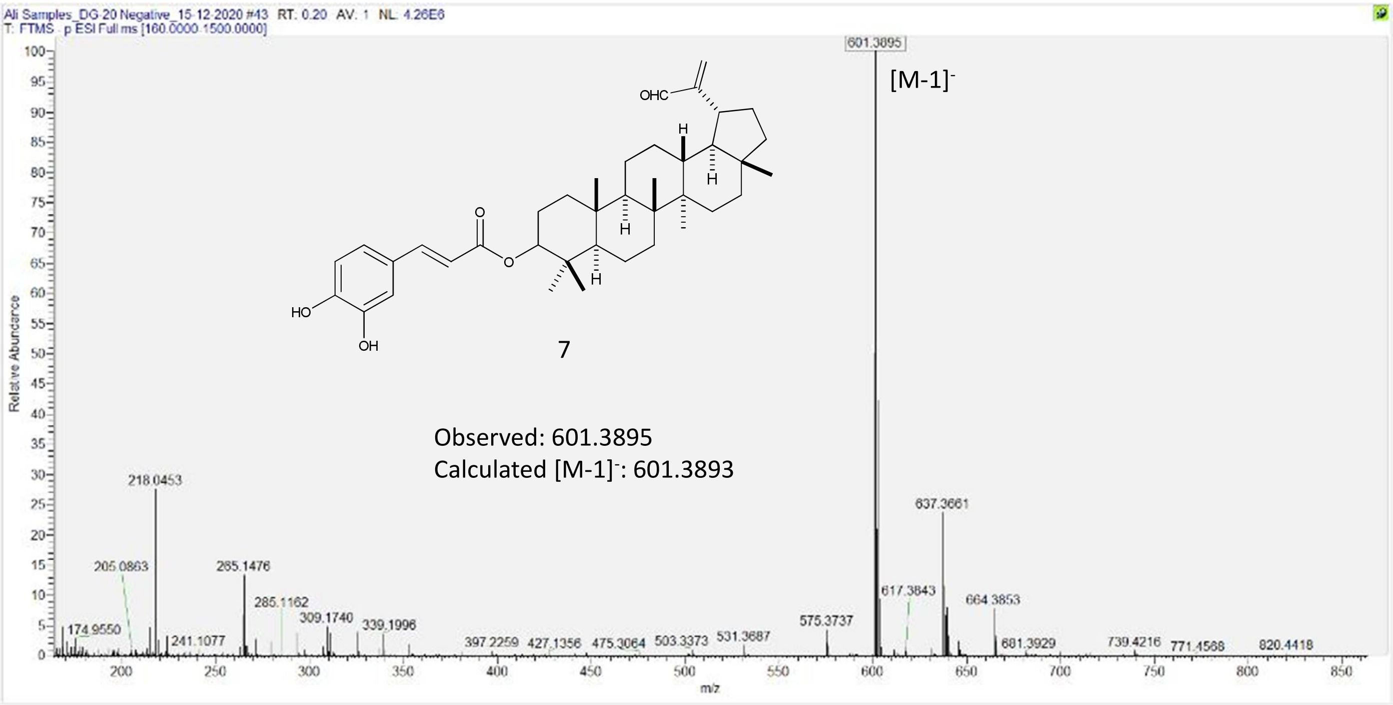

A

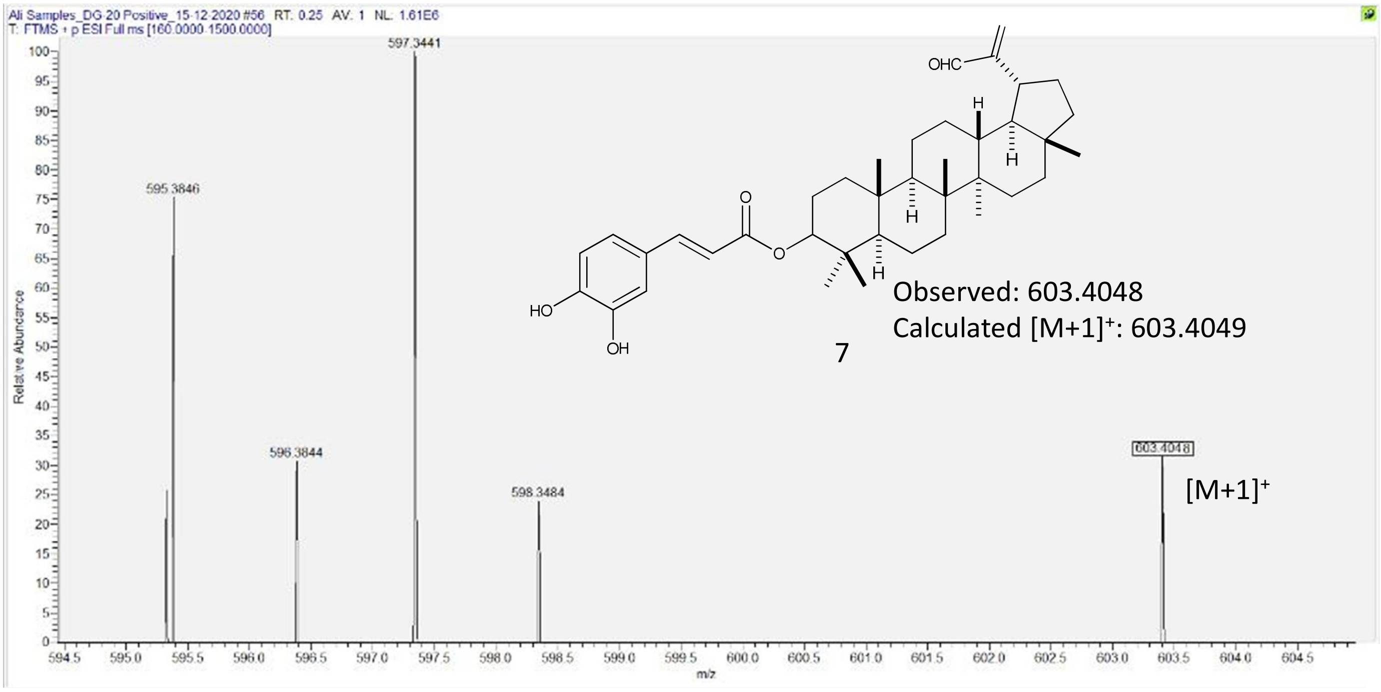

B

Figure 25S: HRESIMS spectrum of compound (**7**) (**A**) negative mode, (**B**) positive mode.

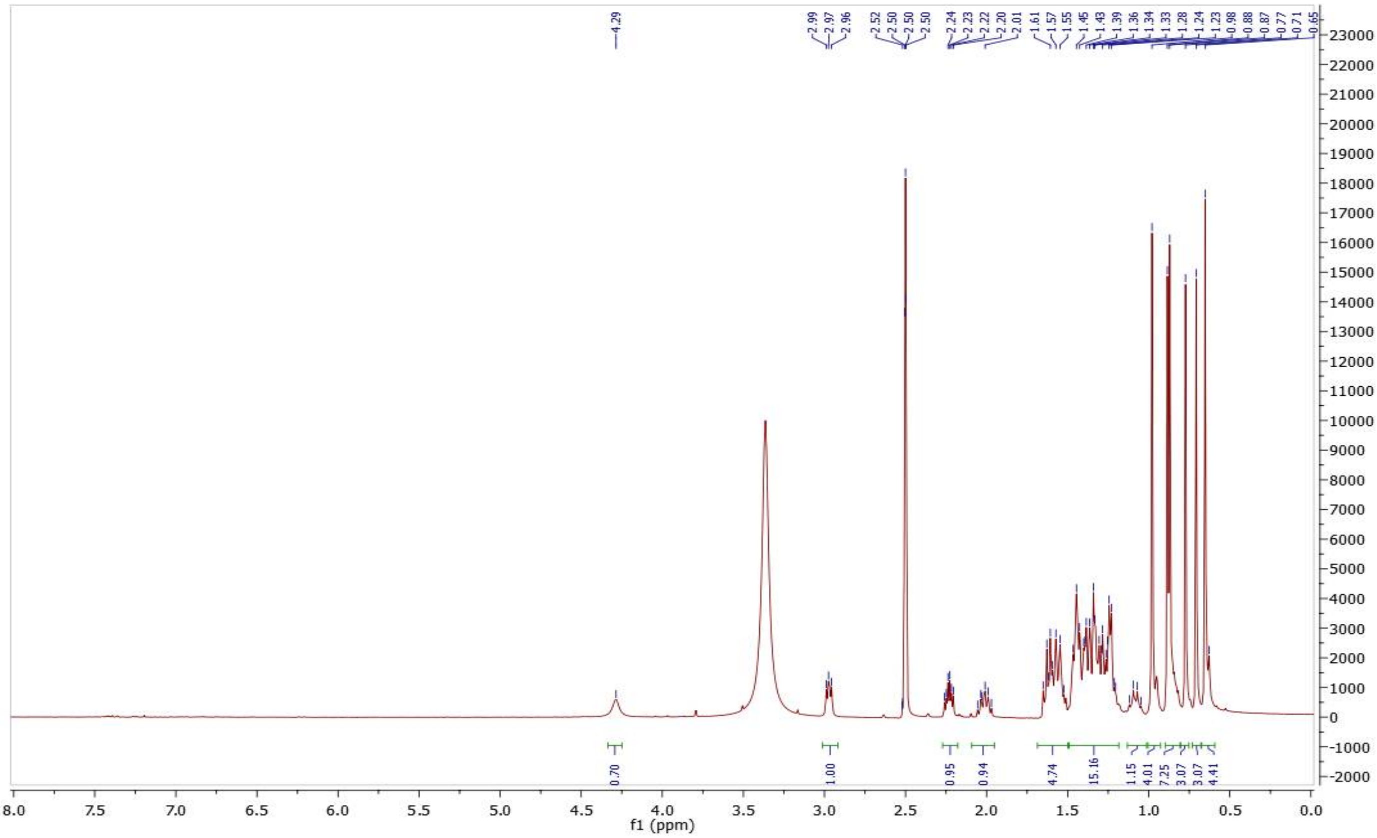

Figure 26S. ^{1}H NMR spectrum of compound (**8**) (500 MHz, $DMSO_6$)

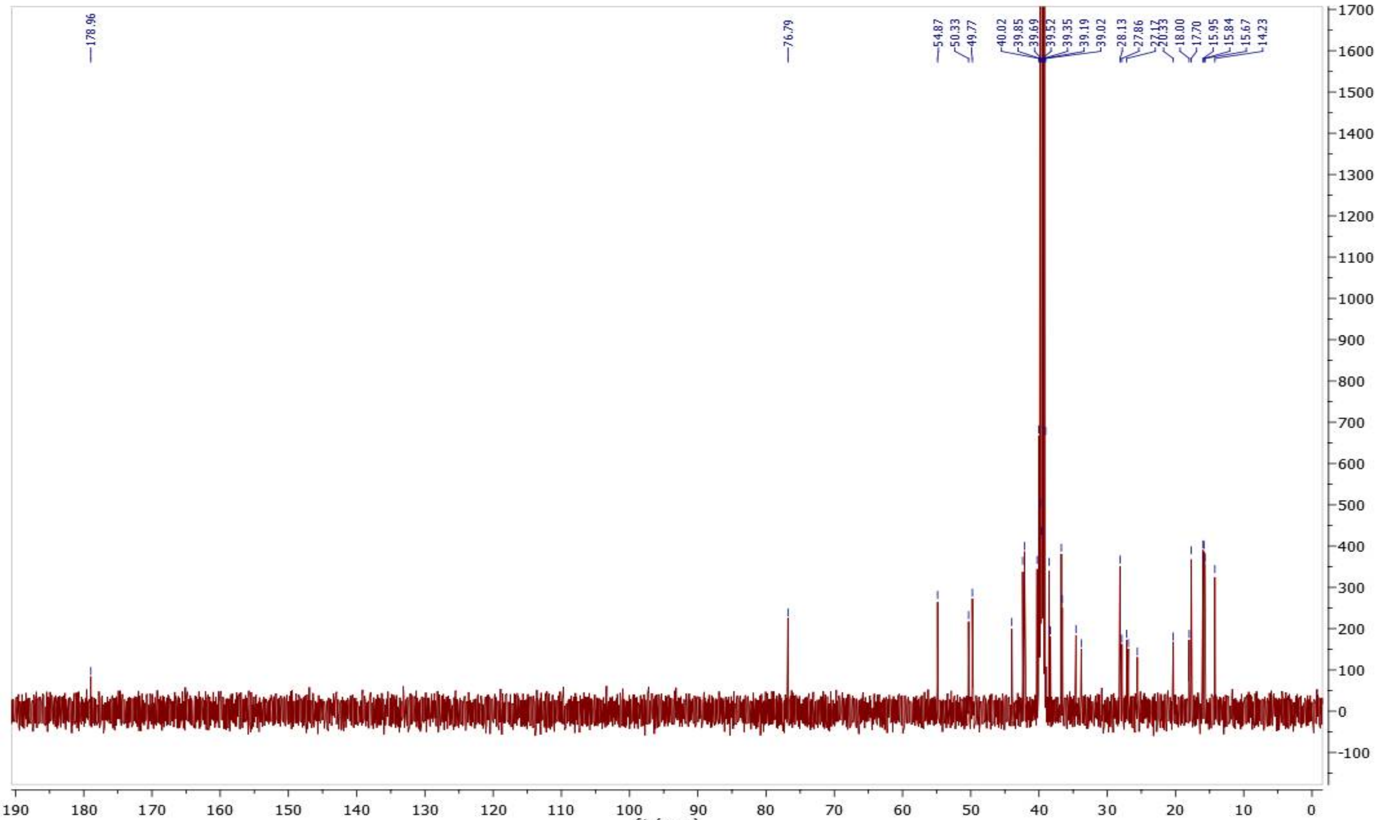

Figure 27S. ^{13}C NMR spectrum of compound (**8**) (125 MHz, $DMSO_6$)

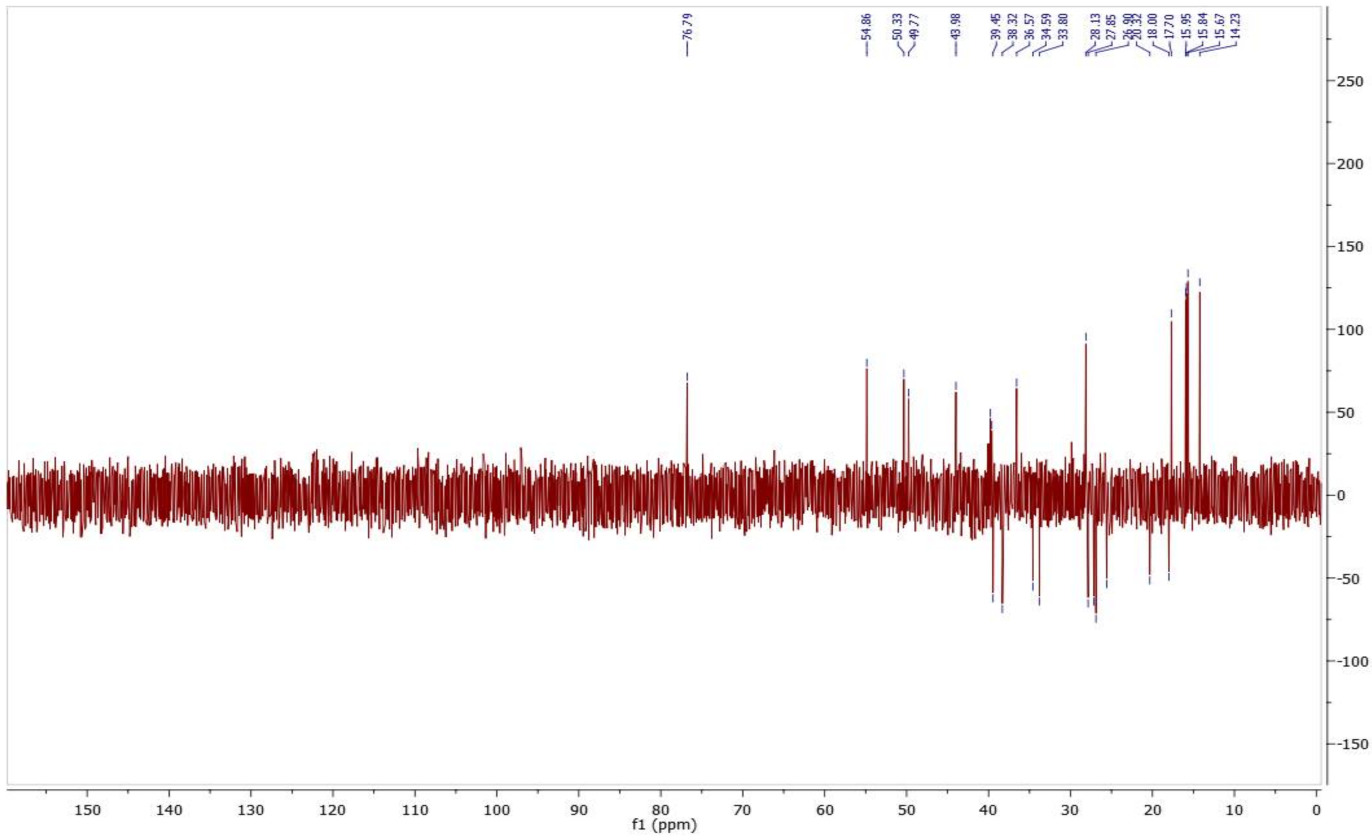

Figure 28S. DEPT ^{13}C NMR spectrum of compound (**8**) (125 MHz, $DMSO_6$)

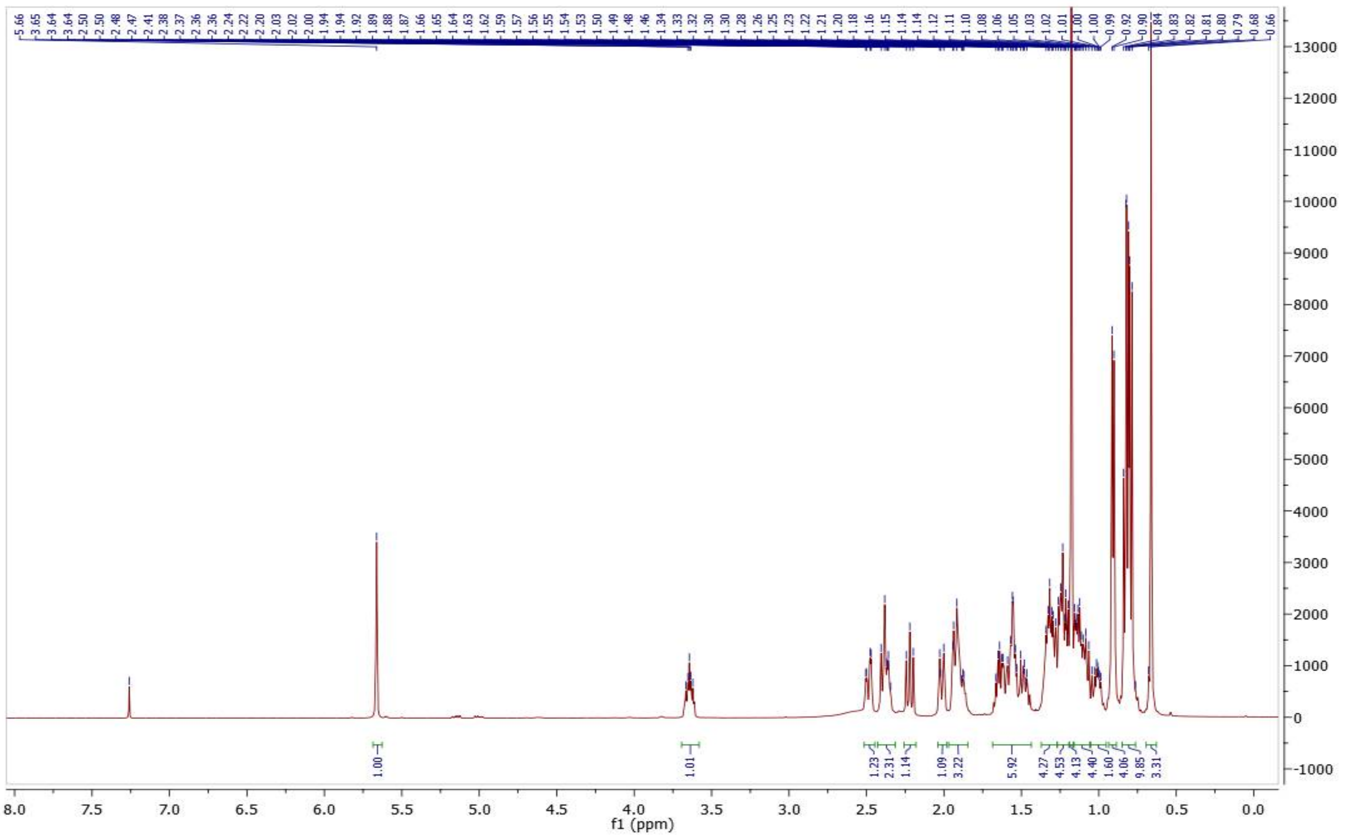

Figure 29S. ^{1}H NMR spectrum of compound (**13**) (500 MHz, $CDCl_3$)

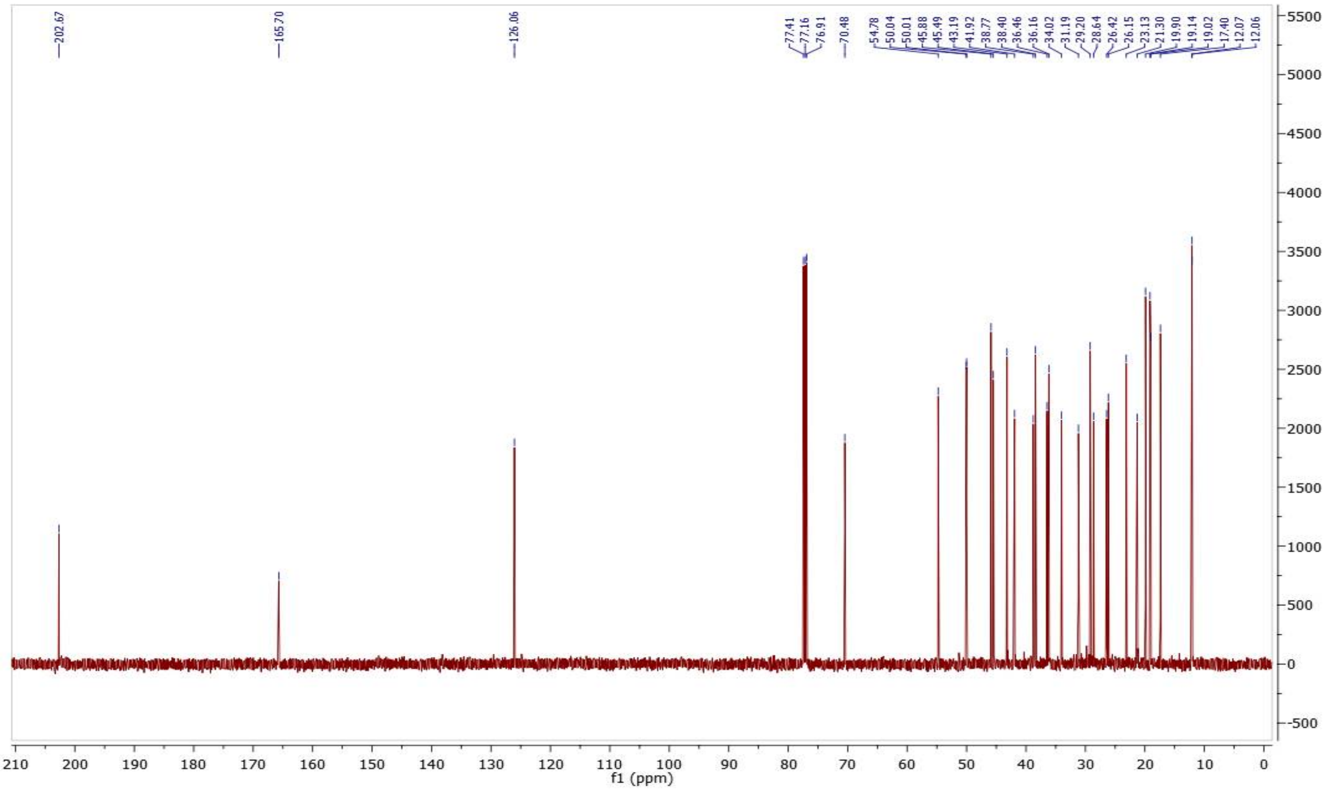

Figure 30S. ^{13}C NMR spectrum of compound (**13**) (125 MHz, $CDCl_3$)

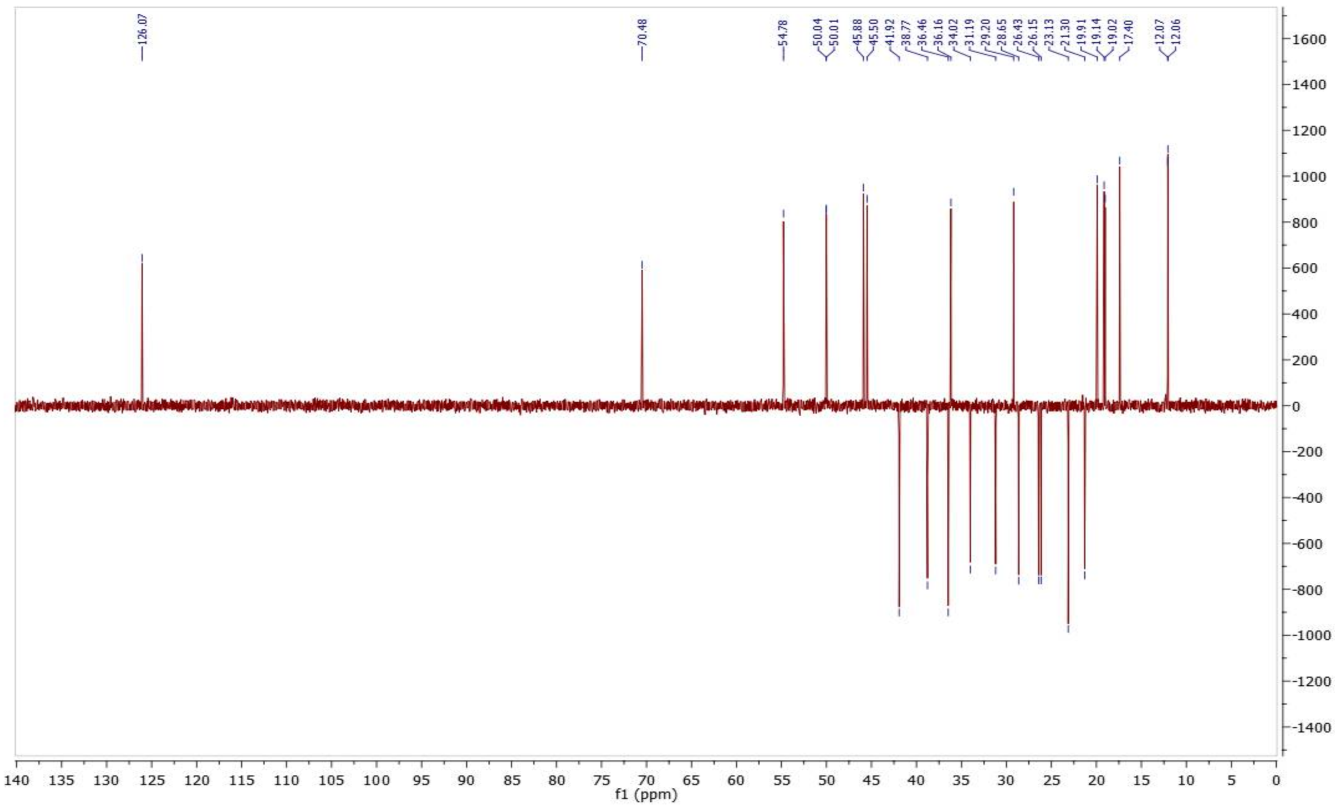

Figure 31S. DEPT ^{13}C NMR spectrum of compound (**13**) (125 MHz, $CDCl_3$)

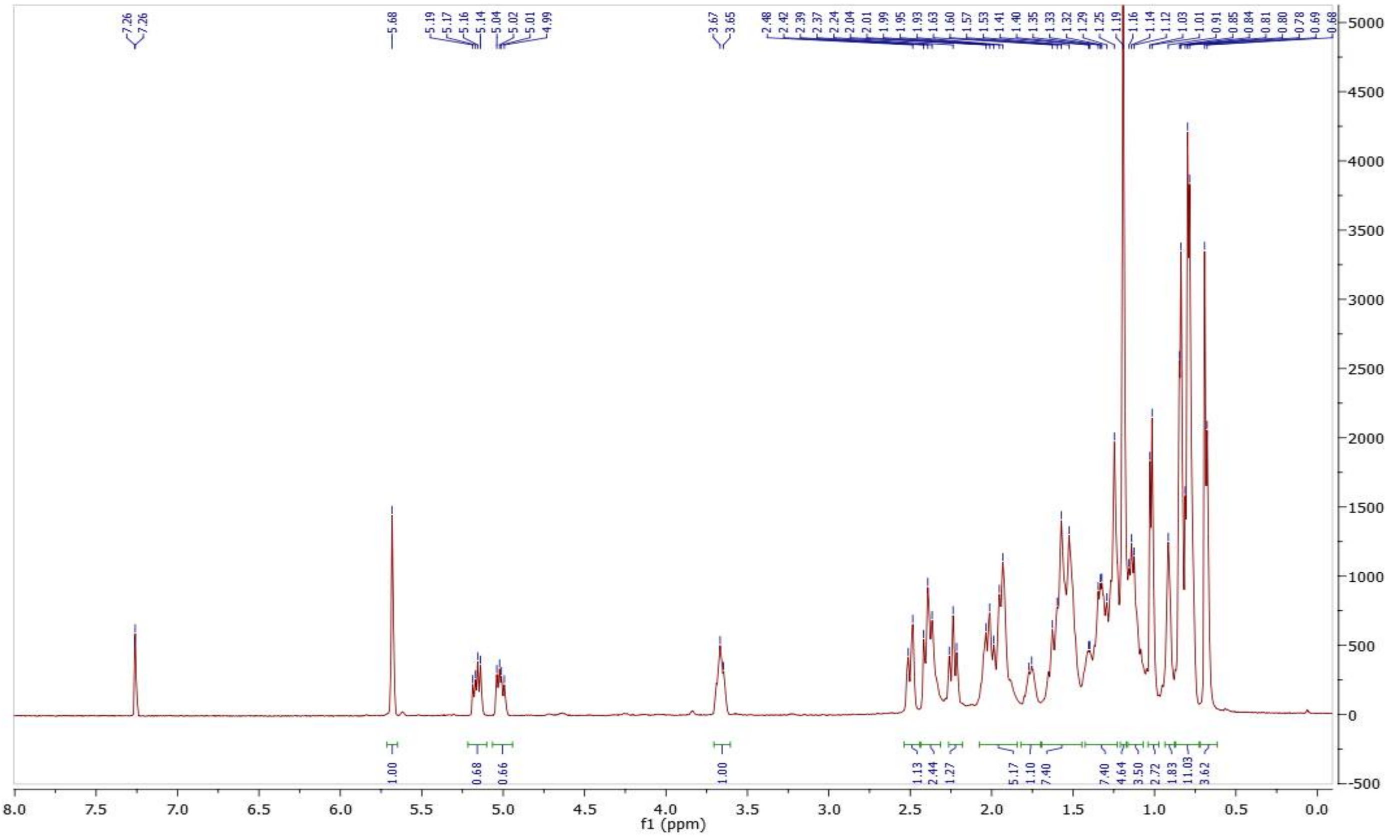

Figure 32S. ^{1}H NMR spectrum of compound (**14**) (500 MHz, $CDCl_3$)

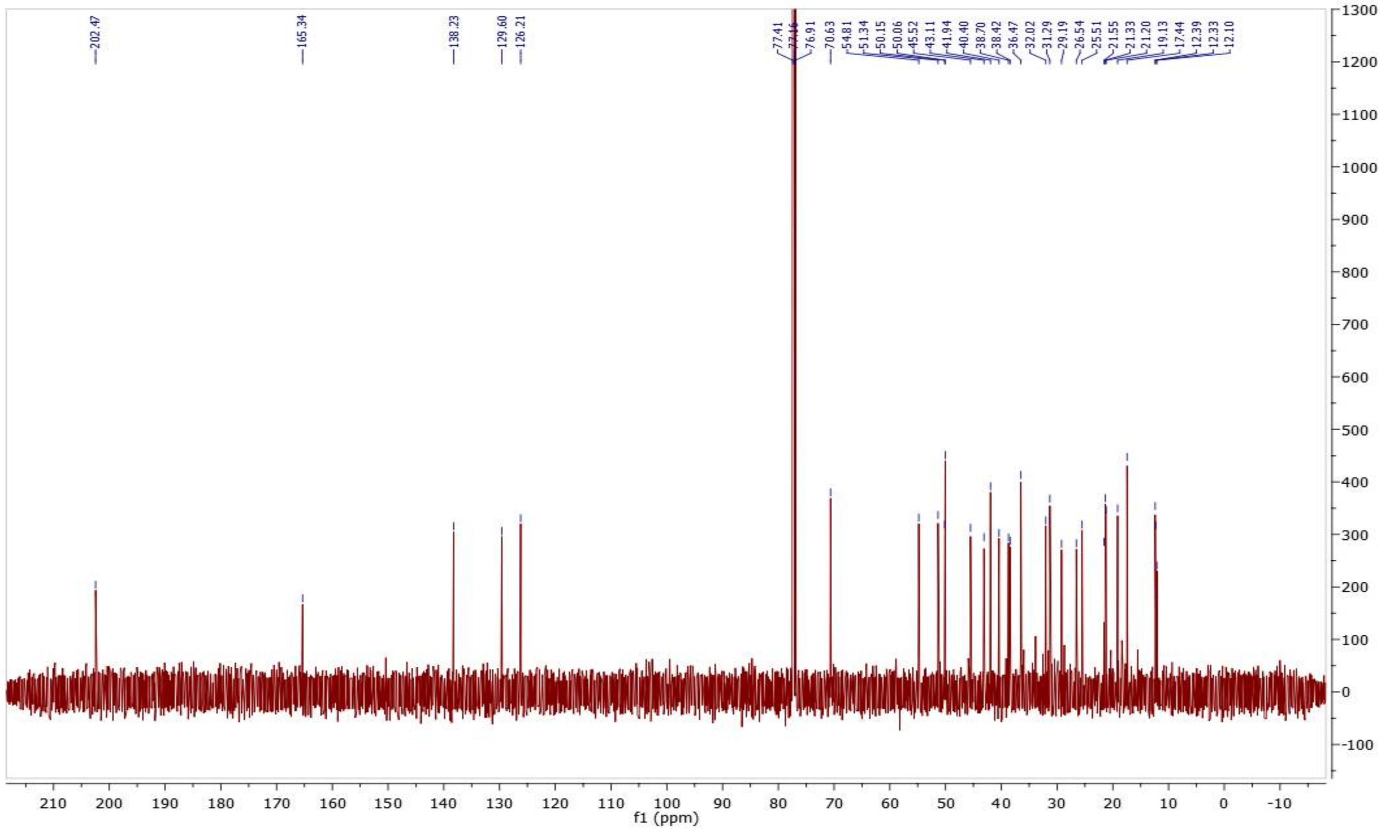

Figure 33S. ^{13}C NMR spectrum of compound (**14**) (125 MHz, $CDCl_3$)

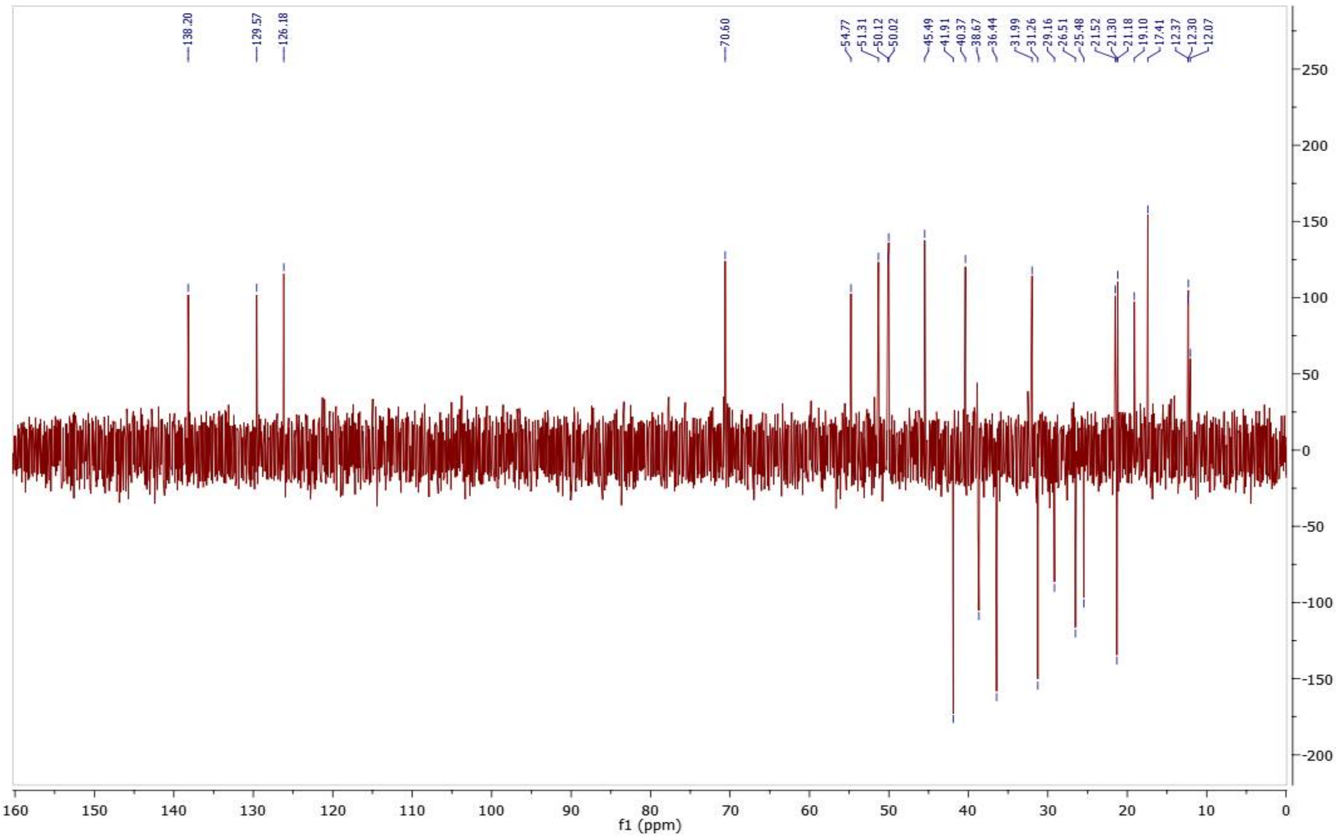

Figure 34S. DEPT ^{13}C NMR spectrum of compound (**14**) (125 MHz, $CDCl_3$)

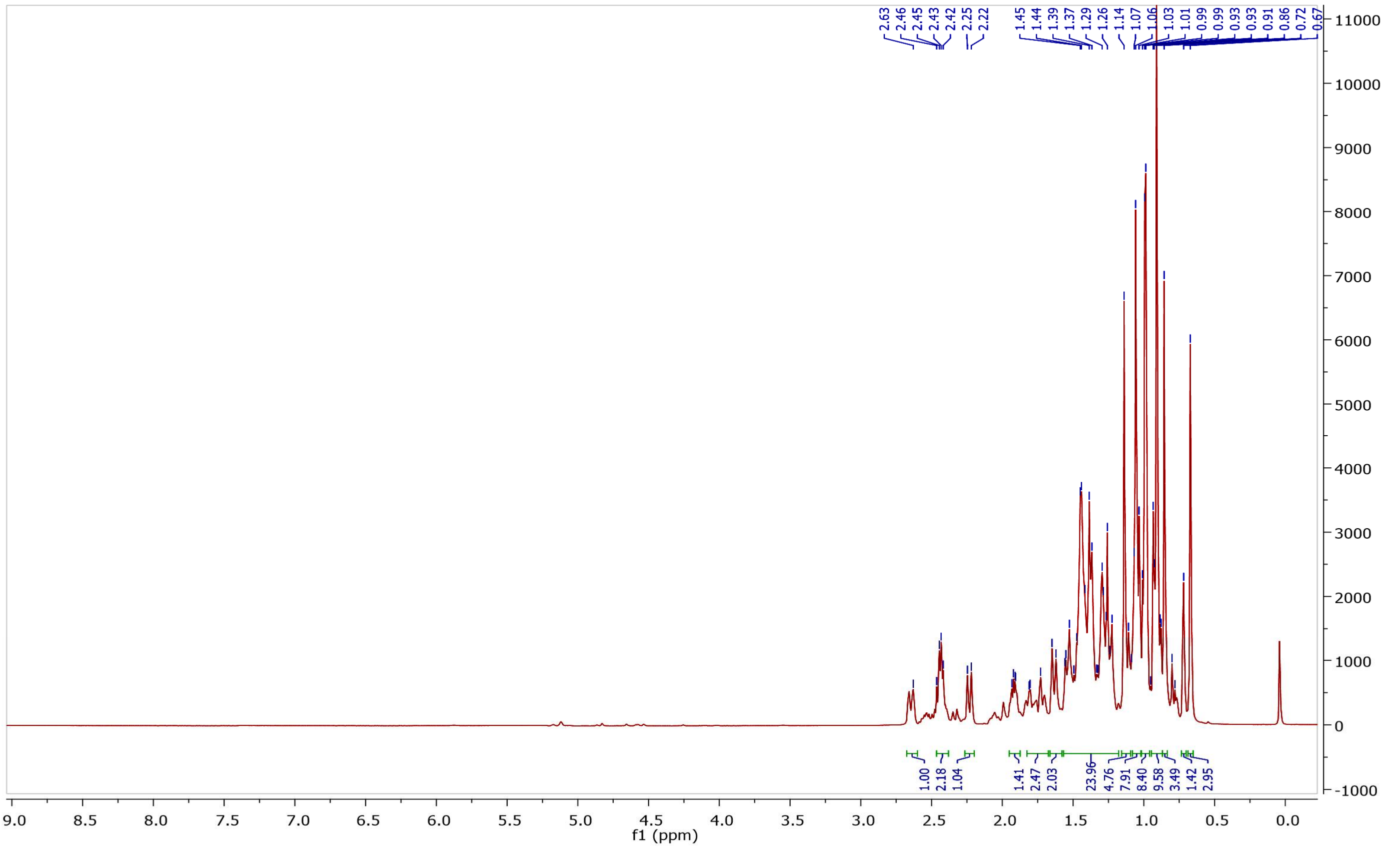

Figure 35S. ^{1}H NMR spectrum of compound (**15**) (500 MHz, $CDCl_3$)

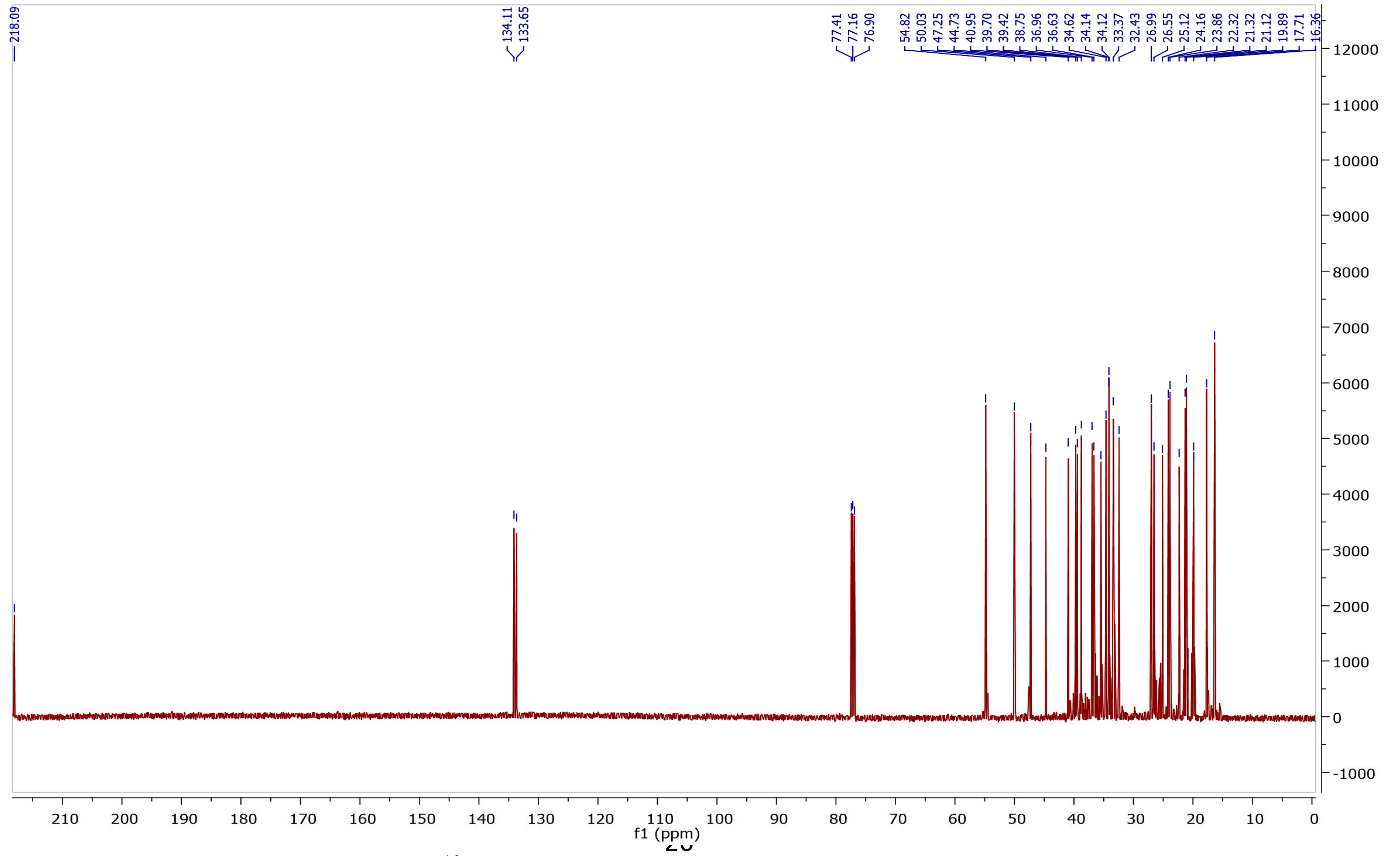

Figure 36S. ^{13}C NMR spectrum of compound (**15**) (125 MHz, $CDCl_3$)

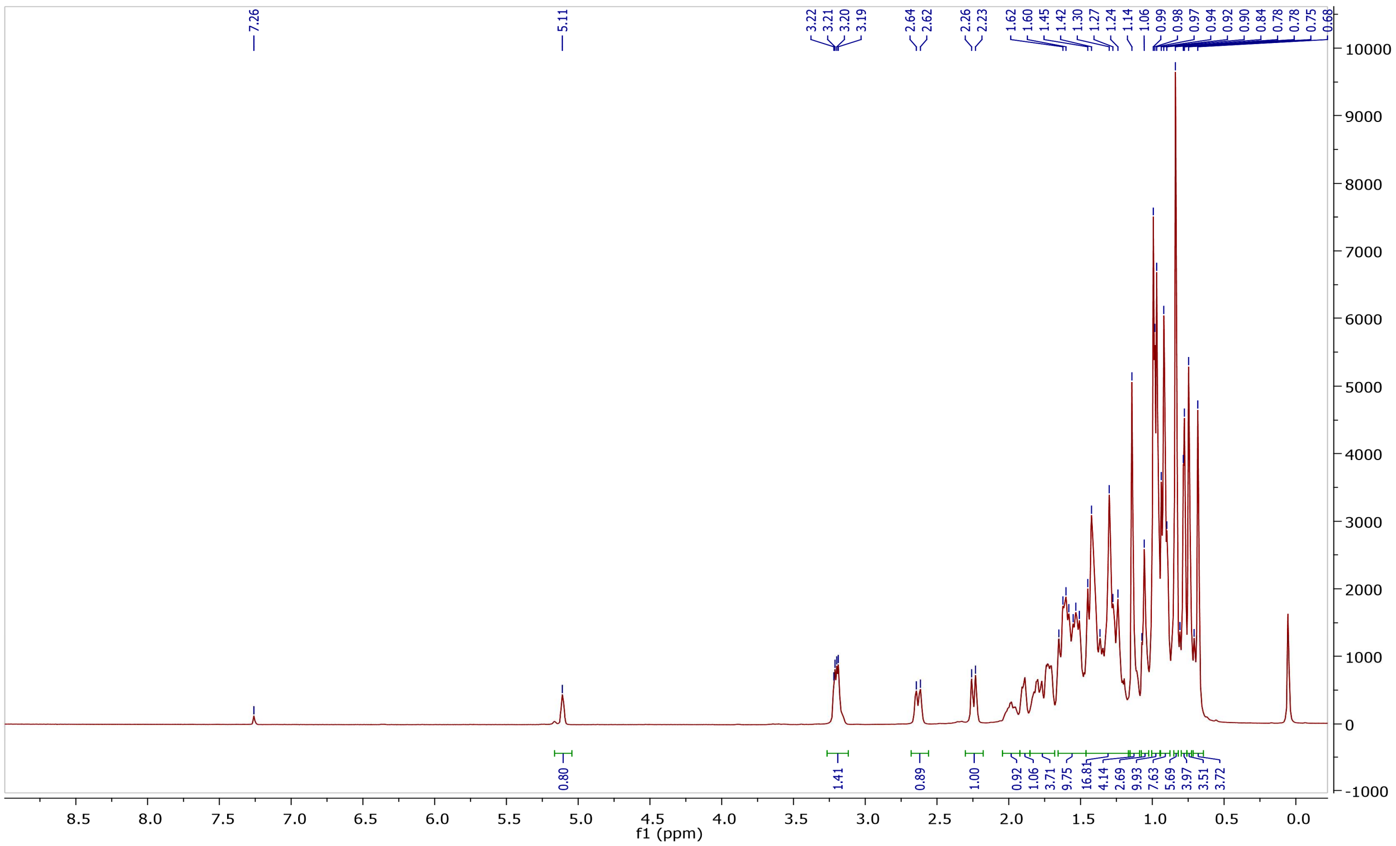

Figure 37S. ^{1}H NMR spectrum of compound (**16**) (500 MHz, $CDCl_3$)

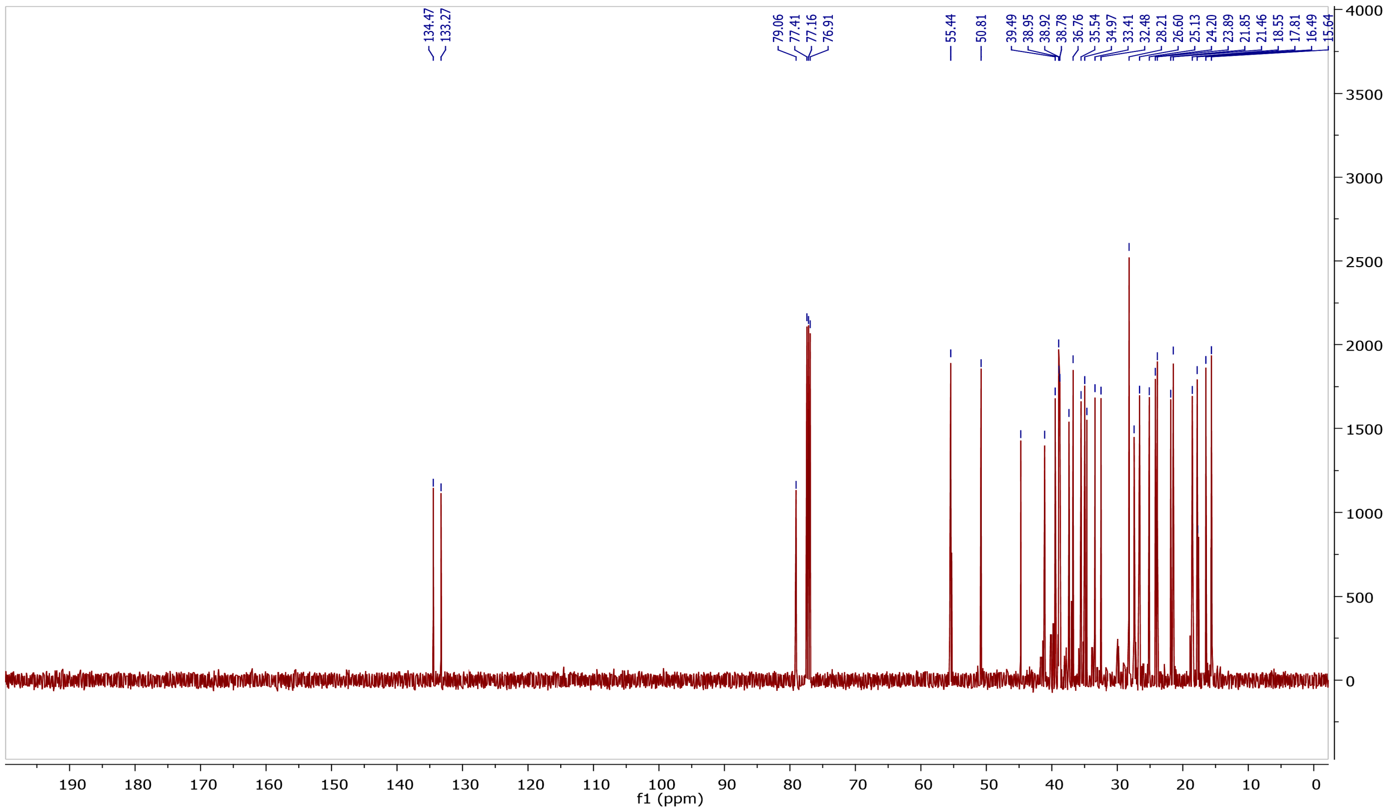

Figure 38S. ^{13}C NMR spectrum of compound (**16**) (125 MHz, $CDCl_3$)

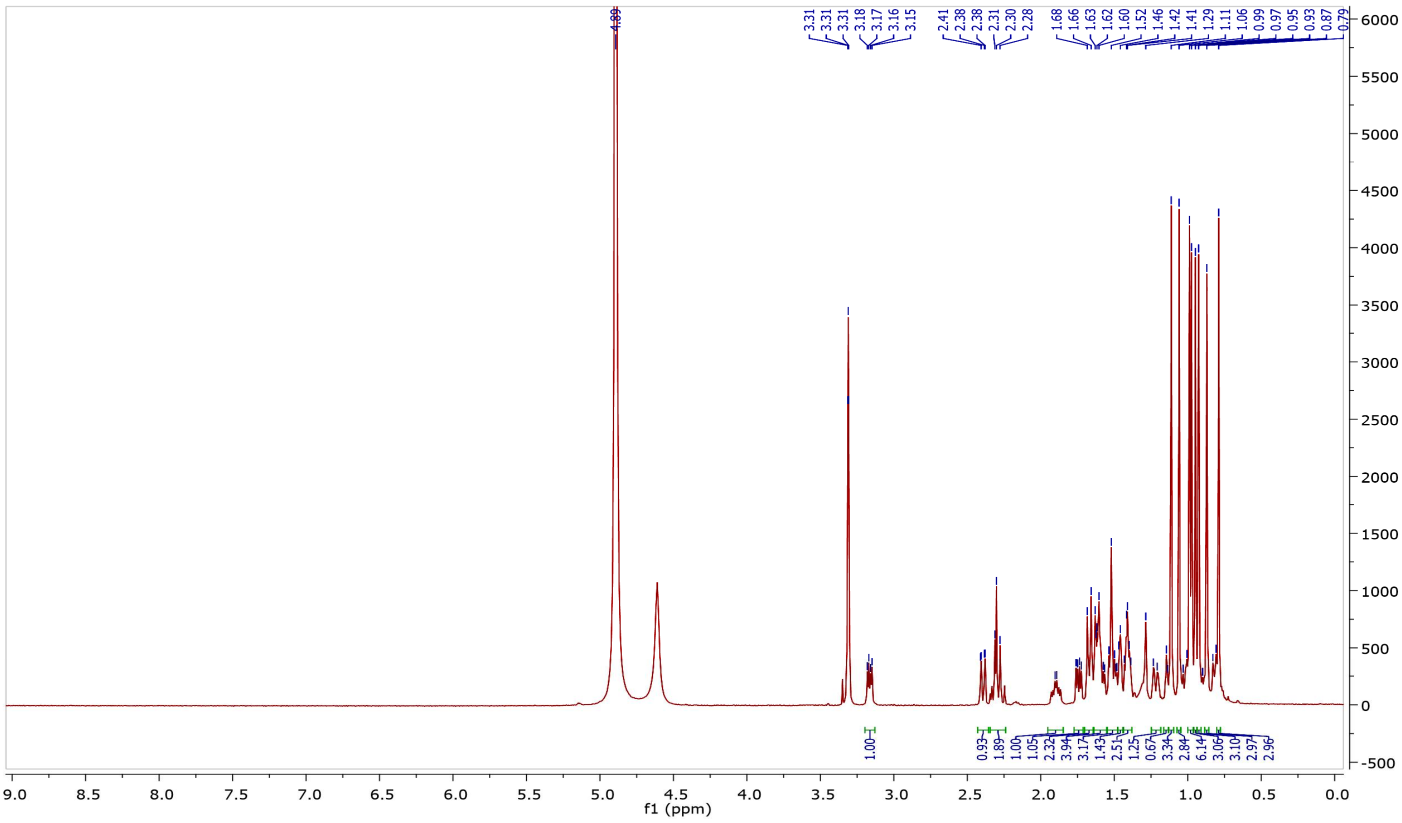

Figure 39S. ^{1}H NMR spectrum of compound (**17**) (500 MHz, CD_3OD)

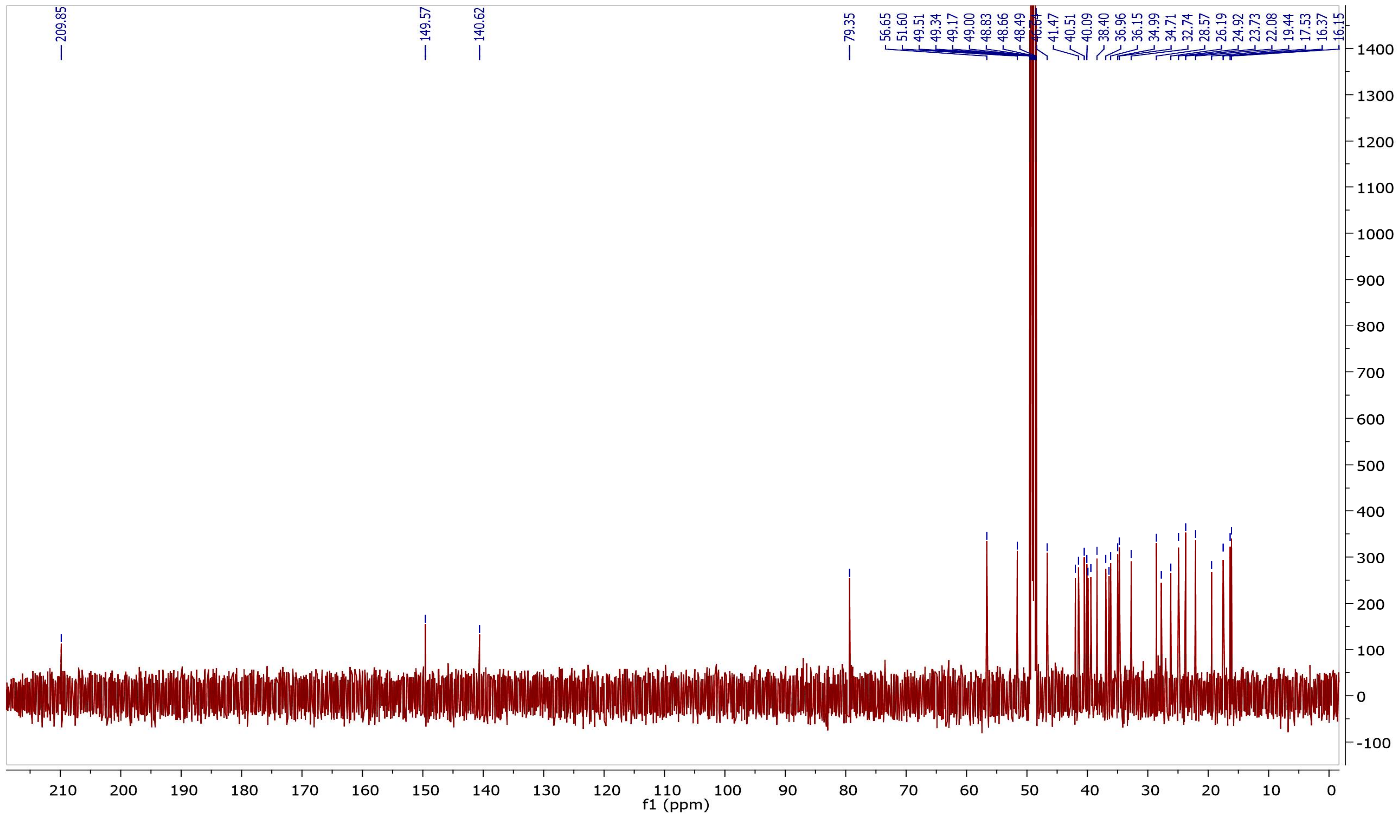

Figure 40S. ^{13}C NMR spectrum of compound (**17**) (125 MHz, CD_3O)

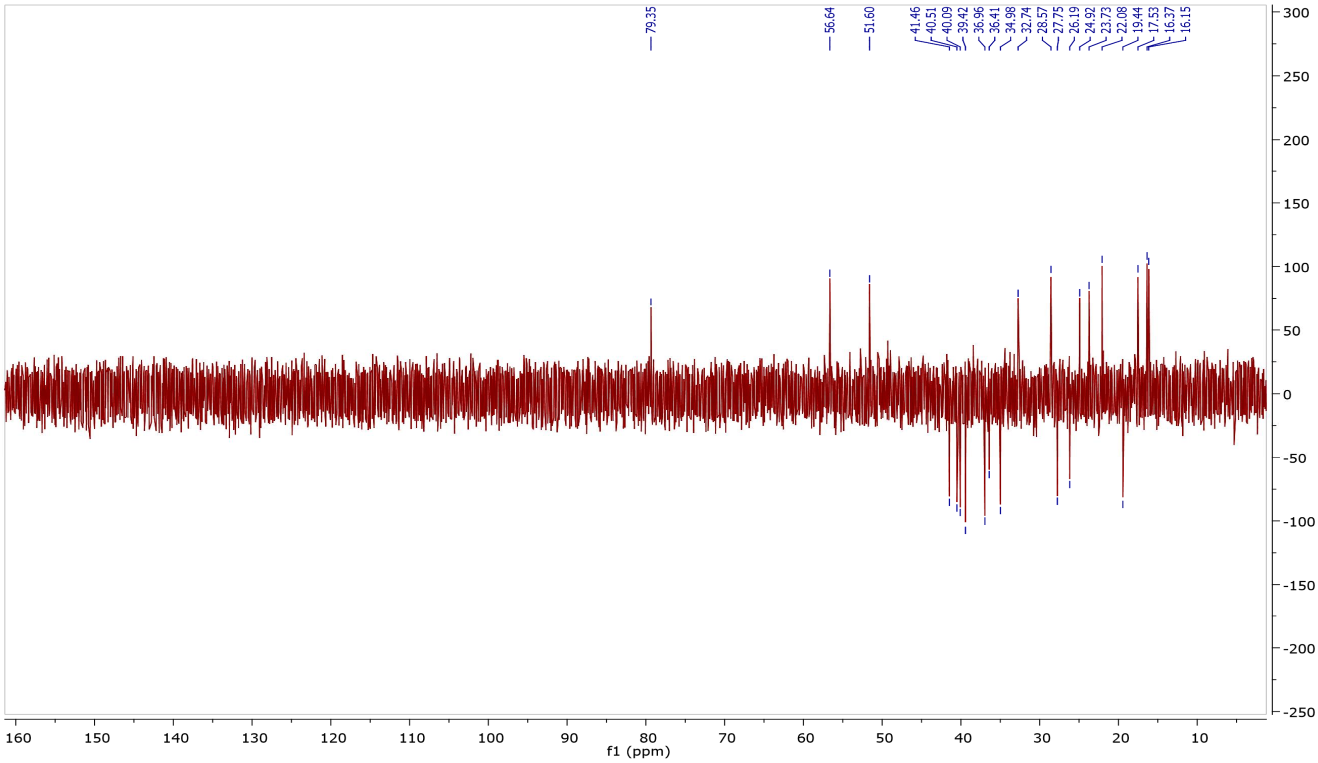

Figure 41S. DEPT ^{13}C NMR spectrum of compound (**17**) (125 MHz, CD_3OD)

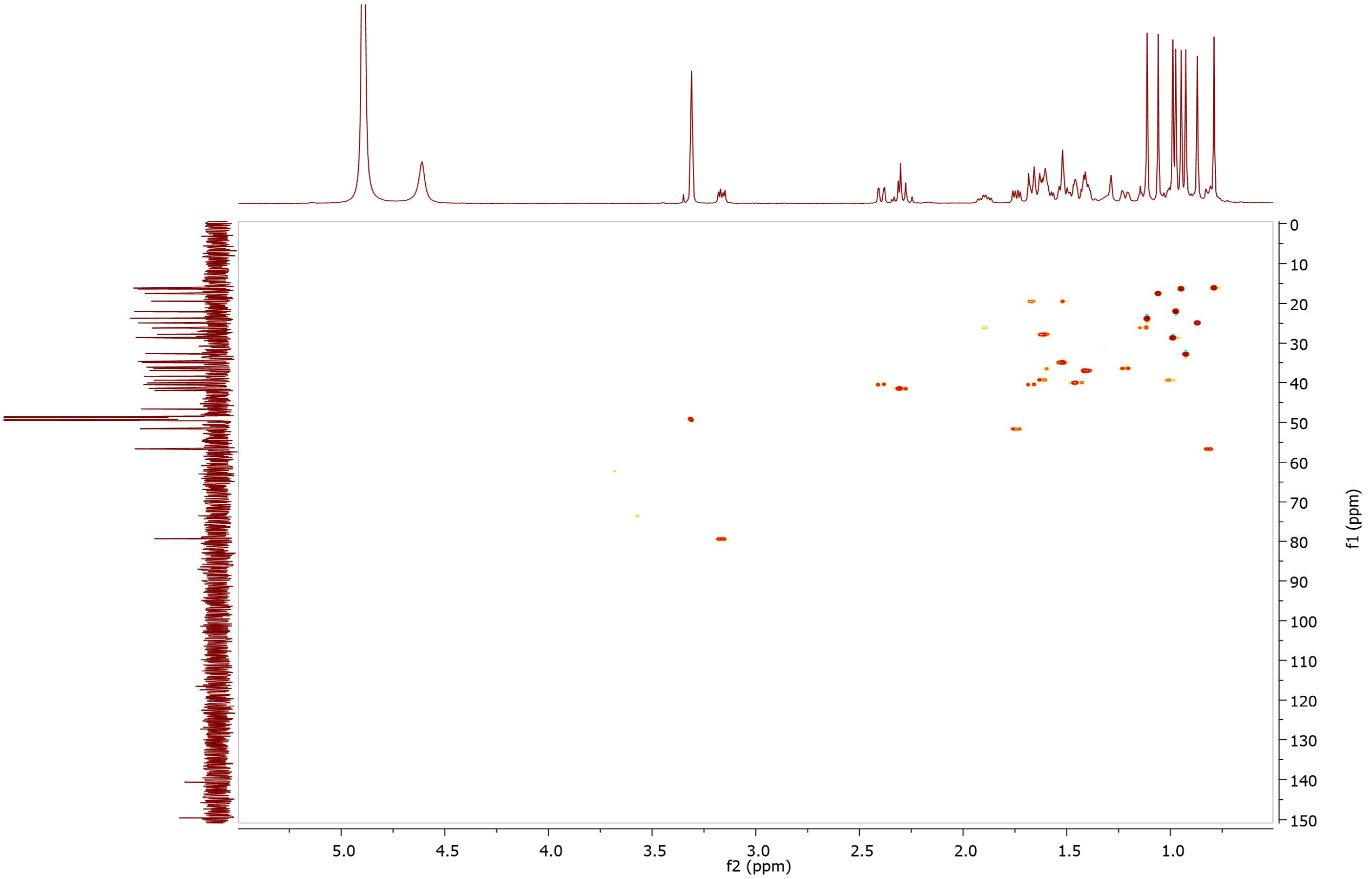

Figure 42S. ^{1}H-^{13}C HSQC spectrum of compound (17) (500 MHz, CD_3OD)

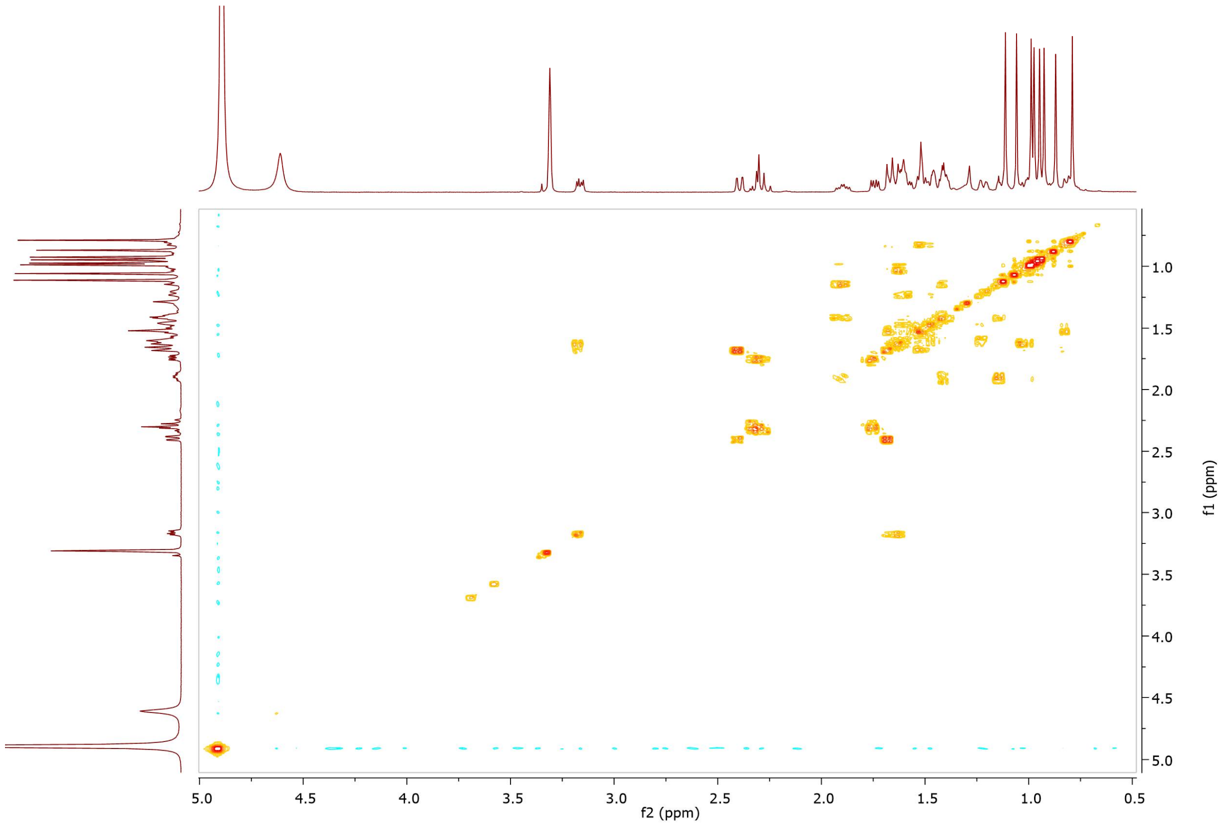

Figure 43S. ^{1}H-^{1}H COSY spectrum of compound (**17**) (500 MHz, CD_3OD)

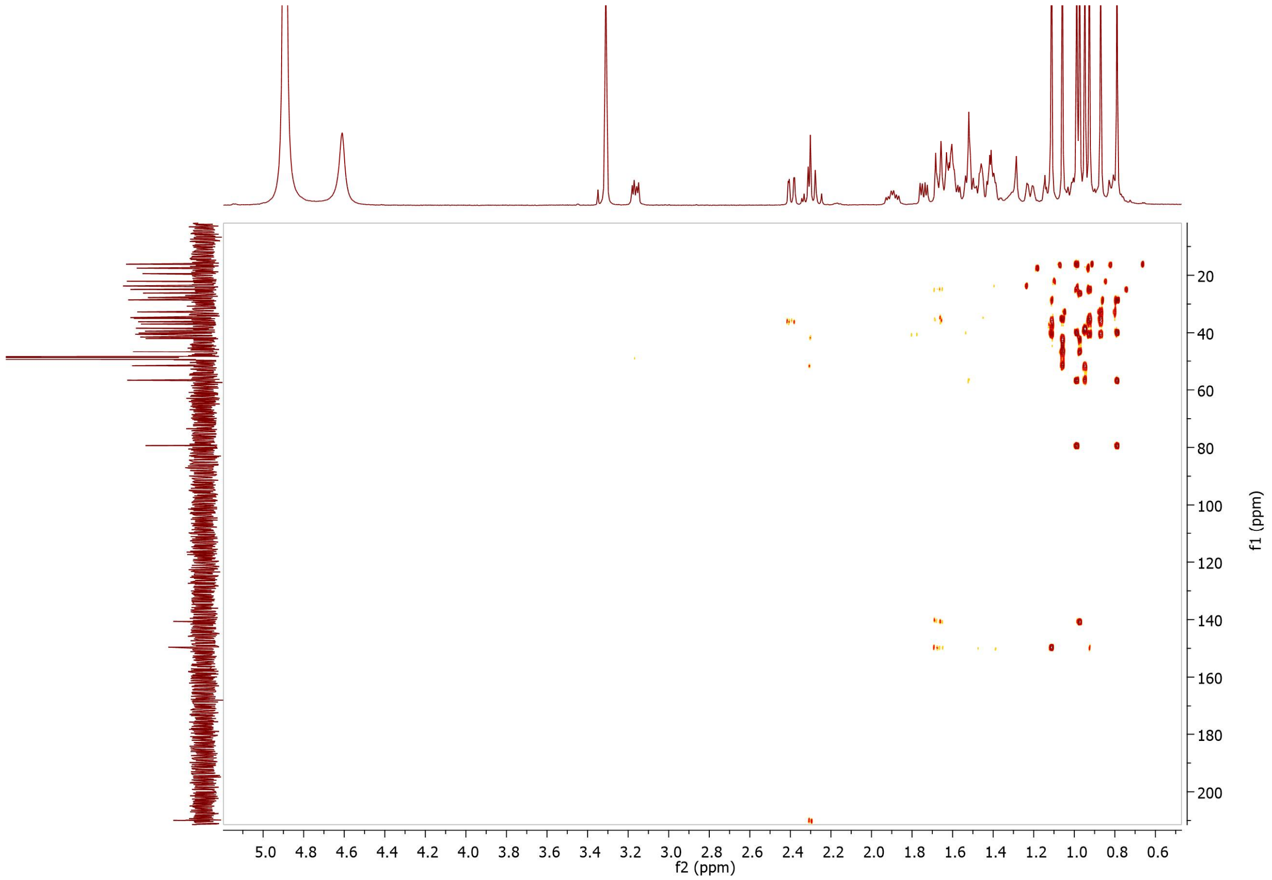

Figure 44S. ^{1}H-^{13}C HMBC spectrum of compound (**17**) (500 MHz, CD_3OD)

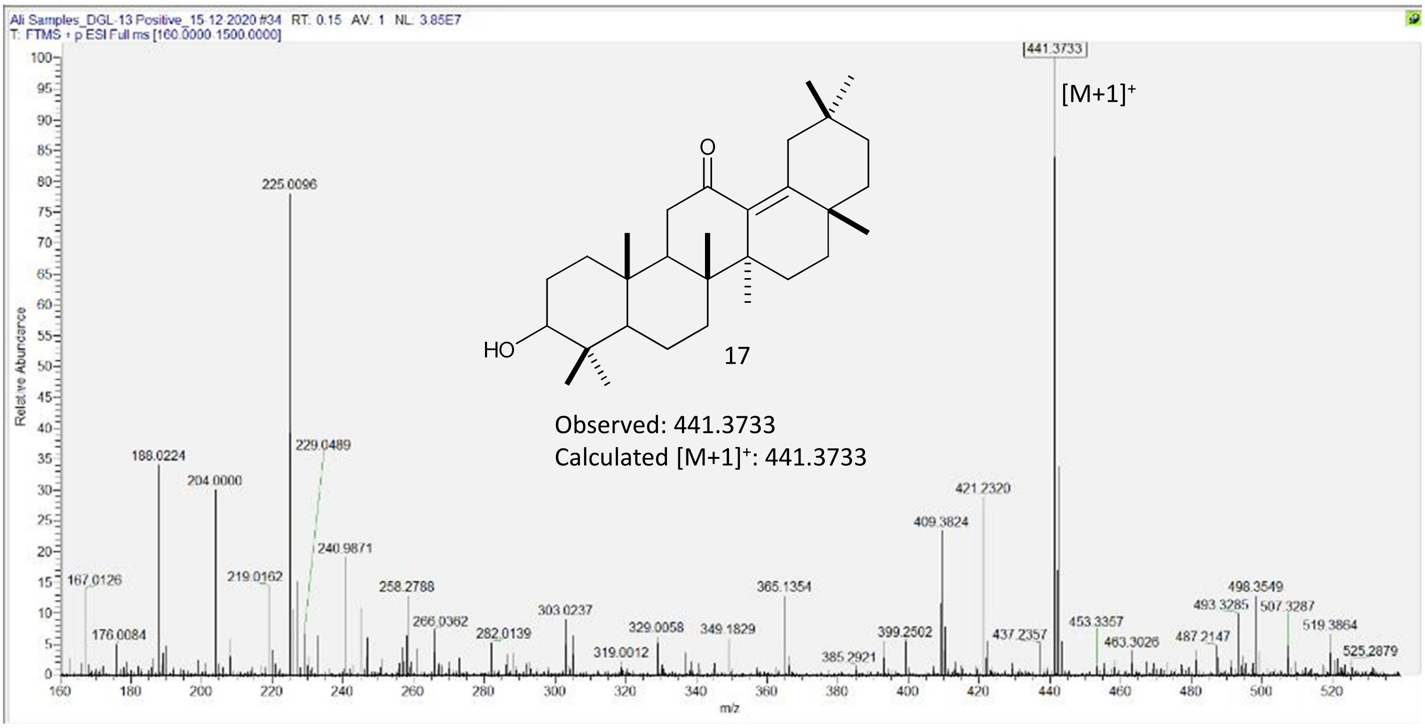

Figure 45S: HRESIMS spectrum of compound (**17**) positive mode.

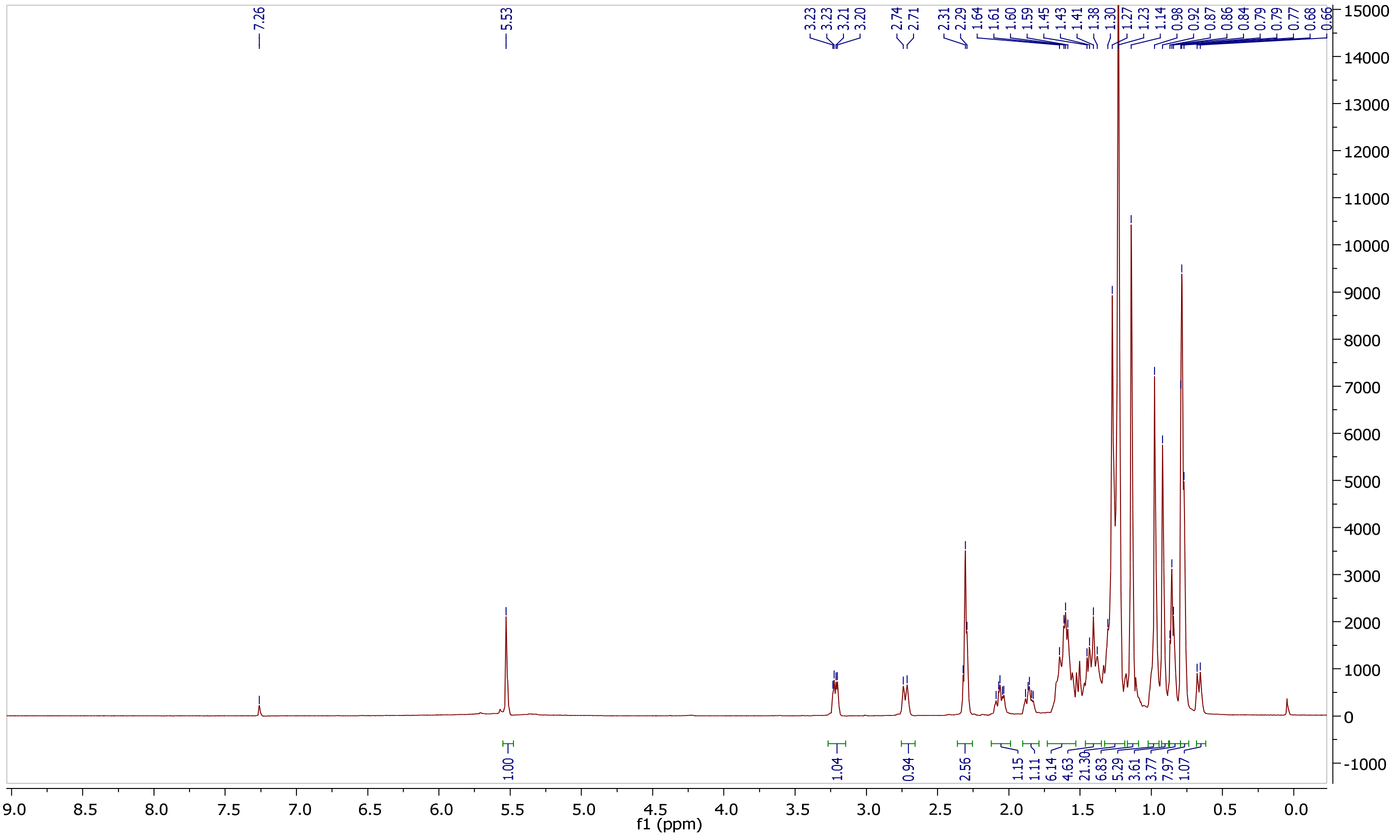

Figure 46S. ^{1}H NMR spectrum of compound (**18**) (500 MHz, $CDCl_3$)

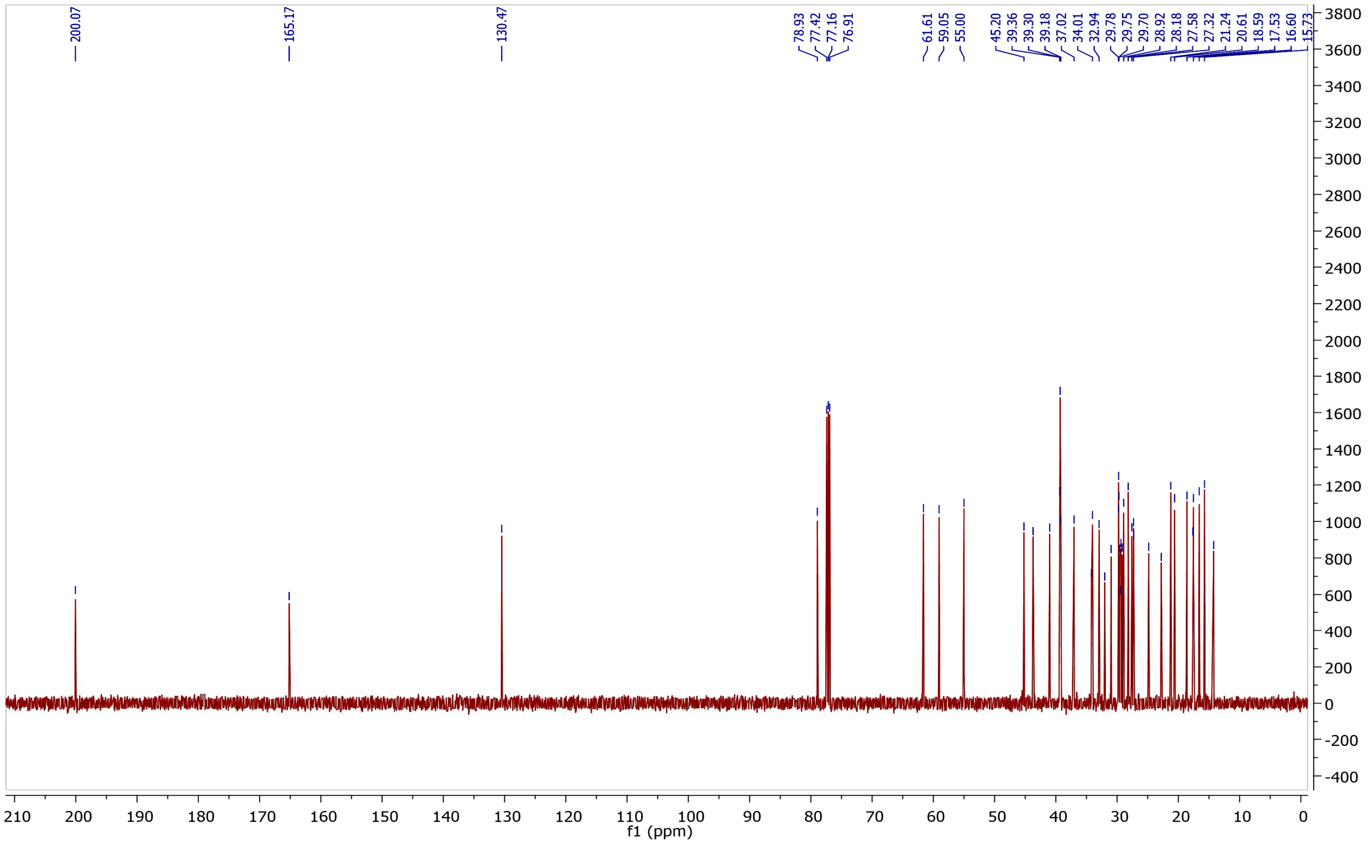

Figure 47S. ^{13}C NMR spectrum of compound (**18**) (125 MHz, $CDCl_3$)

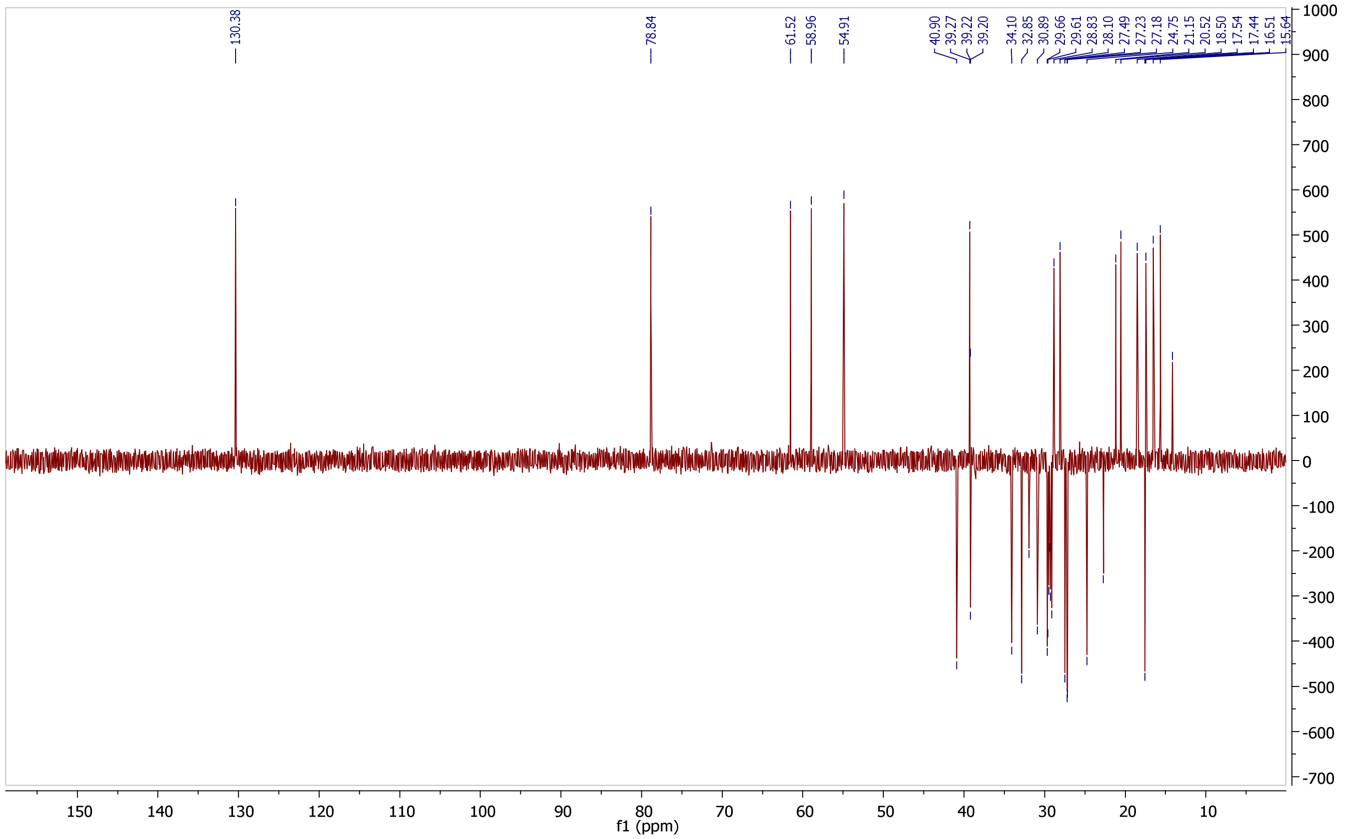

Figure 48S. DEPT ^{13}C NMR spectrum of compound (**18**) (125 MHz, $CDCl_3$)
